# Antibody–drug conjugate development in lymphoma: a systematic clinical trial landscape analysis

**DOI:** 10.3389/fimmu.2026.1855476

**Published:** 2026-08-19

**Authors:** Hengrui Zhang, Jincheng Li, Hanxi Yan, Yulin Liu

**Affiliations:** School of Medical and Life Sciences, Chengdu University of Traditional Chinese Medicine, Chengdu, China

**Keywords:** antibody-drug conjugates (ADCs), clinical trial landscape, linker, lymphoma, payload, target antigen, Trialtrove database

## Abstract

**Introduction:**

Antibody–drug conjugates (ADCs) have established roles in selected lymphoma settings, but the maturity, subtype distribution, and platform diversity of the clinical pipeline remain unclear. We mapped interventional trials to evaluate development trends, evidence maturity, molecular architecture, combination strategies, inactive development, and safety reporting.

**Methods:**

We searched Trialtrove for Phase I–IV interventional trials evaluating at least one ADC in lymphoma, with actual or anticipated start dates through December 31, 2025. Non-ADC studies, observational studies, trials without lymphoma populations, and records with unconfirmed eligibility were excluded. Two reviewers independently screened and classified eligible records, with external verification when available. Analyses were descriptive; treatment effects and patient-level adverse-event incidences were not pooled.

**Results:**

Of 482 records, 410 trials were included. Phase II was the largest category (193, 47.1%); 253 trials (61.7%) were single-arm and 76 (18.5%) were randomized. Among 837 trial–subtype pairs, diffuse large B-cell lymphoma (213, 25.4%) and classical Hodgkin lymphoma (135, 16.1%) were the most frequent and contained most late-phase activity. CD30 (181 trials), CD79b (104), CD19 (47), and CD22 (29) were the leading assigned targets. Of 50 unique ADC programs, 35 (70.0%) had not progressed beyond Phase I or Phase I/II. Tubulin inhibitors and cleavable linkers predominated. Among 92 inactive trials, the leading reported categories were recruitment or feasibility problems (30), unspecified reasons (28), and sponsor strategy (15). In 177 safety-evaluable trials, grade ≥3 adverse events were reported in 109, neutropenia in 98, infection in 79, and peripheral neuropathy in 69.

**Discussion:**

Lymphoma ADC development has expanded, but trial growth has outpaced platform diversification and the generation of confirmatory evidence. Mature development remains concentrated in DLBCL, cHL, and a limited number of target–payload–linker platforms, while randomized, survival-based, and biomarker-driven evidence remains limited. Future priorities include subtype-specific targeting, platform diversification, randomized comparative studies, standardized safety reporting, biomarker-guided patient selection, and rational sequencing with immune and cellular therapies. Trial status and trial-level safety-reporting frequencies should not be interpreted as measures of clinical success or patient-level adverse-event incidence.

## 1 Introduction

Lymphomas comprise Hodgkin lymphoma and a broad group of mature B-cell, T-cell, and natural killer-cell neoplasms. Current classification systems emphasize the marked differences in cellular origin, clinicopathological features, and molecular profiles across these diseases (1, 2). Their incidence and mortality also vary across disease subtypes and geographic regions, but lymphomas remain an important global cancer burden (3, 4). Treatment has improved through risk-adapted chemotherapy, radiotherapy, targeted agents, and immunotherapies. Even so, a clinically important proportion of patients develop relapsed or refractory disease, particularly in aggressive B-cell lymphomas and classical Hodgkin lymphoma (cHL) (5, 6). These patients may require several lines of treatment, which add cumulative toxicity, time burden, and pressure on quality of life (7). New treatments should therefore improve disease control without further increasing the overall treatment burden.

Antibody–drug conjugates (ADCs) provide one approach to this need. An ADC contains three main components: a monoclonal antibody, a chemical linker, and a cytotoxic payload. After the antibody binds to its target antigen, the ADC is usually internalized and processed within the cell, followed by release of the payload and tumor-cell death (8–10). This design can increase tumor-directed drug delivery, but it does not eliminate systemic exposure, off-target effects, or payload-related toxicity (8–10). Clinical trials have established the value of ADCs in several lymphoma settings. Brentuximab vedotin has produced long-term survival benefits in previously untreated stage III or IV cHL and CD30-positive peripheral T-cell lymphoma (11, 12). Polatuzumab vedotin combined with rituximab, cyclophosphamide, doxorubicin, and prednisone improved progression-free survival compared with R-CHOP in previously untreated intermediate- or high-risk diffuse large B-cell lymphoma (DLBCL), and the 5-year POLARIX analysis showed that this benefit was sustained (13, 14). Loncastuximab tesirine has also produced durable responses in patients with relapsed or refractory DLBCL after at least two previous lines of systemic therapy (15).

However, the success of individual agents does not fully describe the broader lymphoma ADC pipeline. The activity and safety of an ADC depend on target expression, antigen accessibility, internalization, linker stability, payload properties, and tumor biology (8–10, 16–18). Antigen heterogeneity, changes in target expression, altered intracellular processing, and payload resistance may reduce treatment sensitivity (17, 18). Agent-specific toxicities can also limit treatment exposure and further development (9, 10, 16, 17). Combination trials, including studies of polatuzumab vedotin with bispecific antibodies, show increasing interest in integrating ADCs with other immune therapies. However, these regimens may add overlapping toxicity and make treatment effects harder to attribute to a single component (16, 19). Existing reviews mainly describe ADC mechanisms, approved products, emerging platforms, or selected hematologic indications (8–10, 16). An earlier clinical-trial landscape analysis examined ADC development across oncology, but it did not resolve the lymphoma pipeline by detailed disease subtype, trial phase, endpoint architecture, target, payload, and linker type (20). Important questions therefore remain about the maturity of lymphoma ADC development, its distribution across subtypes and regions, its reliance on specific platform components, and its ability to generate confirmatory clinical evidence.

To address these questions, we systematically analyzed interventional ADC-related lymphoma trials indexed in Trialtrove with actual or anticipated start dates on or before December 31, 2025. We assessed temporal and geographic trends, funding and sponsor participation, lymphoma subtype, trial phase and status, arm structure, randomization, and primary endpoint architecture. We also evaluated target distributions, payload classes, linker types, combination strategies, ADC program maturity, termination patterns, and reported grade 3 or higher adverse events. Selected landmark and contemporary trials were used to place these development patterns in clinical context, but trial-level efficacy and safety outcomes were not pooled. This study aimed to identify areas with mature evidence and areas with limited subtype coverage, platform diversity, or confirmatory trial development.

## 2 Methods

### 2.1 Study design, data source, and search strategy

We conducted a systematic landscape analysis of registered interventional clinical trials evaluating antibody–drug conjugates (ADCs) in lymphoma. The study characterized temporal and geographic patterns, lymphoma subtype distribution, trial design, endpoint selection, ADC platform characteristics, inactive development, toxicity reporting, and the developmental maturity of individual ADC programs. It was not designed to estimate pooled treatment effects.

Trial records were retrieved from the Trialtrove database through the Citeline Clinical Intelligence platform, which compiles information from international and national trial registries, publications, conference materials, regulatory sources, and sponsor-reported updates. The complete retrieval strategy was:

(“Drug Type: Antibody–drug conjugate”) AND (“Disease: Oncology: Lymphoma, Hodgkin’s” OR “Disease: Oncology: Lymphoma, Non-Hodgkin’s”) AND (“Actual Start Date ≤ 2025/12/31” OR “Anticipated Start Date ≤ 2025/12/31”) AND (“Trial Phase: I, I/II, II, II/III, III, III/IV, or IV”).

The two disease filters represented the parent Hodgkin and non-Hodgkin lymphoma categories in the Trialtrove hierarchy. Individual lymphoma subtypes were identified and standardized during manual data extraction rather than searched separately. No restrictions were applied by country, sponsor type, or trial status. Both actual and anticipated start dates were included; start year was defined by the actual start date when available and by the anticipated date for planned trials without an actual start date.

The database search and data export were completed on 20 January 2026. The prespecified trial-start cutoff was 31 December 2025. Study identification, selection, and reporting were guided by the PRISMA 2020 statement. A completed PRISMA 2020 checklist is provided as a separate **Supplementary File**. Because this study was a systematic clinical-trial landscape analysis rather than a meta-analysis of intervention effects, items that were not applicable and methods that were not undertaken are identified and justified in the checklist. The review protocol was not prospectively registered.

### 2.2 Eligibility criteria and study selection

Eligible studies were interventional Phase I–IV trials that evaluated at least one ADC and enrolled patients with Hodgkin lymphoma, non-Hodgkin lymphoma, or a defined lymphoma subtype. For eligibility, an ADC was defined as an antibody covalently conjugated through a chemical linker to a pharmacologically active payload. Initial identification was based on the Trialtrove ADC filter, followed by manual confirmation using the trial record and external sources where available. Records in which the investigational intervention could not be confirmed as an ADC were excluded. Trials were excluded if they did not involve an ADC, did not include patients with lymphoma, were non-interventional, or had ADC exposure or lymphoma eligibility that could not be confirmed.

Mixed hematologic-malignancy trials were retained when patients with lymphoma were eligible or a lymphoma-specific cohort could be identified. Trials involving acute lymphoblastic leukemia and lymphoblastic lymphoma were included only when lymphoblastic lymphoma involvement was confirmed. Multi-arm studies were counted as one trial in trial-level analyses.

Duplicate records were assessed using trial identifiers, sponsor protocol numbers, and study titles; no duplicate trial records were identified. Two reviewers independently screened all records, with disagreements resolved by consensus. Missing payload, linker, endpoint, sponsor, or other platform information did not itself result in exclusion. Unconfirmed variables were coded as “undisclosed”, “not reported” or “unclear”.

### 2.3 Data extraction, verification, and classification

Two reviewers extracted trial identifier, ADC name, title, start year, phase, status, location, sponsor and collaborator information, lymphoma subtype, target, primary payload and payload class, primary linker type, regimen, arm structure, randomization, primary endpoints, result availability, toxicity reporting, and prior treatment exposure when explicitly reported.

Trialtrove served as the primary data source. Information was externally verified, where available, using public trial registries, peer-reviewed publications, regulatory documents, and official sponsor materials. When sources differed, the most specific verifiable source was used; unresolved variables were not inferred.

Sponsor types followed Trialtrove categories: Top-20 pharmaceutical companies, other pharmaceutical companies, academic institutions, government agencies, and cooperative groups. The Top-20 category was retained as defined by Trialtrove and was not independently re-ranked.

Lymphoma subtypes were standardized as classical Hodgkin lymphoma, nodular lymphocyte-predominant Hodgkin lymphoma, diffuse large B-cell lymphoma, follicular lymphoma, mantle cell lymphoma, marginal zone lymphoma or extranodal mucosa-associated lymphoid tissue lymphoma, chronic lymphocytic leukemia or small lymphocytic lymphoma, lymphoblastic lymphoma or acute lymphoblastic leukemia overlap, anaplastic large cell lymphoma, peripheral T-cell lymphoma, cutaneous T-cell lymphoma, Waldenström macroglobulinemia or lymphoplasmacytic lymphoma, primary mediastinal B-cell lymphoma, high-grade B-cell lymphoma, other B-cell malignancies, and unspecified or mixed lymphoma populations.

All confirmed ADC targets within a trial were retained. For trial-level payload and linker analyses, one primary ADC platform was assigned to each trial, defined as the principal investigational ADC identified in the trial record and confirmed through external sources when necessary. When no principal ADC could be distinguished, the platform was coded as unclear.

Payloads were recorded individually and grouped as tubulin inhibitors, DNA-damaging agents, topoisomerase I inhibitors, RNA-directed agents, other classes, or undisclosed. Exploratory toxicity analyses additionally used MMAE, MMAF, calicheamicin-derived, pyrrolobenzodiazepine-related, and maytansinoid platform families. Linkers were classified as cleavable, non-cleavable, or undisclosed; further chemical subclassification was not performed because linker details were inconsistently reported.

A unique ADC program was defined as a distinct ADC drug or development code. Alternative names and sponsor-specific codes for the same ADC were consolidated, whereas different ADCs directed against the same target were treated as separate programs.

Primary endpoints were classified as safety-related endpoints, dose-limiting toxicity, maximum tolerated dose, recommended Phase II dose or recommended dose for expansion, pharmacokinetics, immunogenicity, objective response rate or response rate, complete response or complete metabolic response, duration of response, progression-free survival, overall survival, other time-to-event or relapse endpoints, minimal residual disease, biomarker or correlative endpoints, other response or progression endpoints, feasibility or transplant-related endpoints, and other or unclear assessments.

“Any safety-related endpoint” was a composite category encompassing adverse events, toxicity, tolerability, dose-limiting toxicity, maximum tolerated dose, vital signs, laboratory abnormalities, and organ-specific safety measures. DLT and MTD were also coded separately and therefore overlapped with the broader safety category. Trials with multiple primary endpoint types contributed to each applicable category.

Non-ADC combination partners were grouped as chemotherapy or corticosteroids, monoclonal antibodies or other immunotherapies, targeted small molecules or immunomodulatory agents, immune checkpoint inhibitors, bispecific antibodies or T-cell engagers, cellular therapy or transplant-related strategies, and other or unidentified agents. These categories were not mutually exclusive.

Treatment setting and line of therapy were not used as core quantitative variables because their definitions were incomplete and inconsistent across sources. Prior treatment exposure was retained only when explicitly reported and was used for contextual safety analyses.

Selected landmark and contemporary studies were used to illustrate the evolution of lymphoma ADC development. Selection emphasized pivotal or practice-informing completed trials and recent late-phase programs across major subtypes and platforms. Study-level efficacy and safety findings were verified from primary publications, regulatory documents, and public registries and were not pooled. The complete trial-level analytical dataset is provided in **Supplementary Table 1**. The classification and coding rules used to construct the analytical variables are described in Methods Sections 2.2–2.5 and the corresponding figure legends. **Supplementary Table 2** provides additional details for the selected landmark and contemporary trials.

### 2.4 Units of analysis and denominators

The individual clinical trial was the primary analytical unit, with 410 eligible trials included.

For the regional analysis, participating countries were mapped to six continental regions: Africa, Asia, Europe, North America, Oceania, and South America. Each trial was assigned to one mutually exclusive continent or continent-combination category. Country-level mapping was conducted separately using trial–country records; therefore, multinational trials contributed to more than one country on the choropleth map; lymphoma subtype analyses used trial–subtype pairs; target analyses used target–trial records; and sponsor involvement was assessed using mutually exclusive trial-level funding categories and non-mutually exclusive sponsor–trial records.

Overall endpoint frequencies were calculated at the trial level. Phase-stratified endpoint analyses used trial–endpoint category records, whereas subtype-stratified analyses used trial–subtype–endpoint category records. Trials could therefore contribute to multiple records when they included several subtypes, endpoint categories, or both.

Overall payload and linker analyses were based on the primary ADC platform, with each trial contributing no more than one payload and one linker assignment. Subtype-stratified analyses used trial–subtype–payload and trial–subtype–linker records. Subtype-stratified target analyses used trial–subtype–target records.

Program-level developmental maturity was assessed by assigning each unique ADC program to the highest clinical phase attained in lymphoma trials. This was not interpreted as a phase-transition rate or probability of developmental success. Because analytical units differed, some totals exceeded the 410 unique trials. The unit and denominator for each analysis were specified in the corresponding figure legend or table footnote.

### 2.5 Trial status, inactive development, and safety reporting

Trialtrove status labels were standardized as open, completed, terminated, planned, closed, or temporarily closed. Completed status was considered an operational outcome and not evidence of clinical benefit. No trial was excluded on the basis of development status. Trial status was assigned according to the information available on the database export date of 20 January 2026. Trials without an actual start date were retained as planned trials when an anticipated start date on or before 31 December 2025 was available. Suspended or withdrawn records, when present in the underlying sources, were retained under the corresponding standardized Trialtrove status rather than excluded. The standardized status assigned to each included trial is provided in **Supplementary Table 1**.

Terminated, closed, and temporarily closed trials were analyzed as inactive-status records. Explicitly reported reasons were categorized as recruitment or feasibility problems, sponsor strategy or pipeline reprioritization, insufficient efficacy, safety or adverse effects, funding problems, administrative or other reasons, no further development reported, or no specific reason available. Reasons were not inferred from target, payload, linker, or sponsor type.

Safety analyses included trials with available results reporting at least one usable toxicity outcome by event type, grade, frequency, or clinical consequence. Categories included grade ≥3 adverse events, neutropenia, anemia, thrombocytopenia, infection, peripheral neuropathy, hepatotoxicity, ocular toxicity, pneumonitis or interstitial lung disease, toxicity-related discontinuation, dose modification, and treatment-related death.

Because adverse-event definitions, populations, regimens, follow-up, and reporting formats varied, safety outcomes were summarized as the number and proportion of safety-evaluable trials reporting each category. Patient-level adverse-event incidence was not pooled.

### 2.6 Statistical analysis

Categorical variables were summarized as counts and percentages. Temporal patterns were assessed by trial-start year and predefined time periods. All analyses used the prespecified units and denominators described above.

No formal hypothesis testing or pooled treatment-effect estimation was performed; therefore, p-values were not used. Formal risk-of-bias and certainty-of-evidence assessments were not applied because registered trials were included irrespective of result availability and comparative treatment effects were not synthesized. No sensitivity analysis was prespecified because no pooled or model-based estimate was generated.

Data were organized in Microsoft Excel 2019. Descriptive analyses and figures were produced using Python.

## 3 Results

### 3.1 Temporal, geographic, and sponsor patterns of lymphoma ADC trials

**Figure 1:**
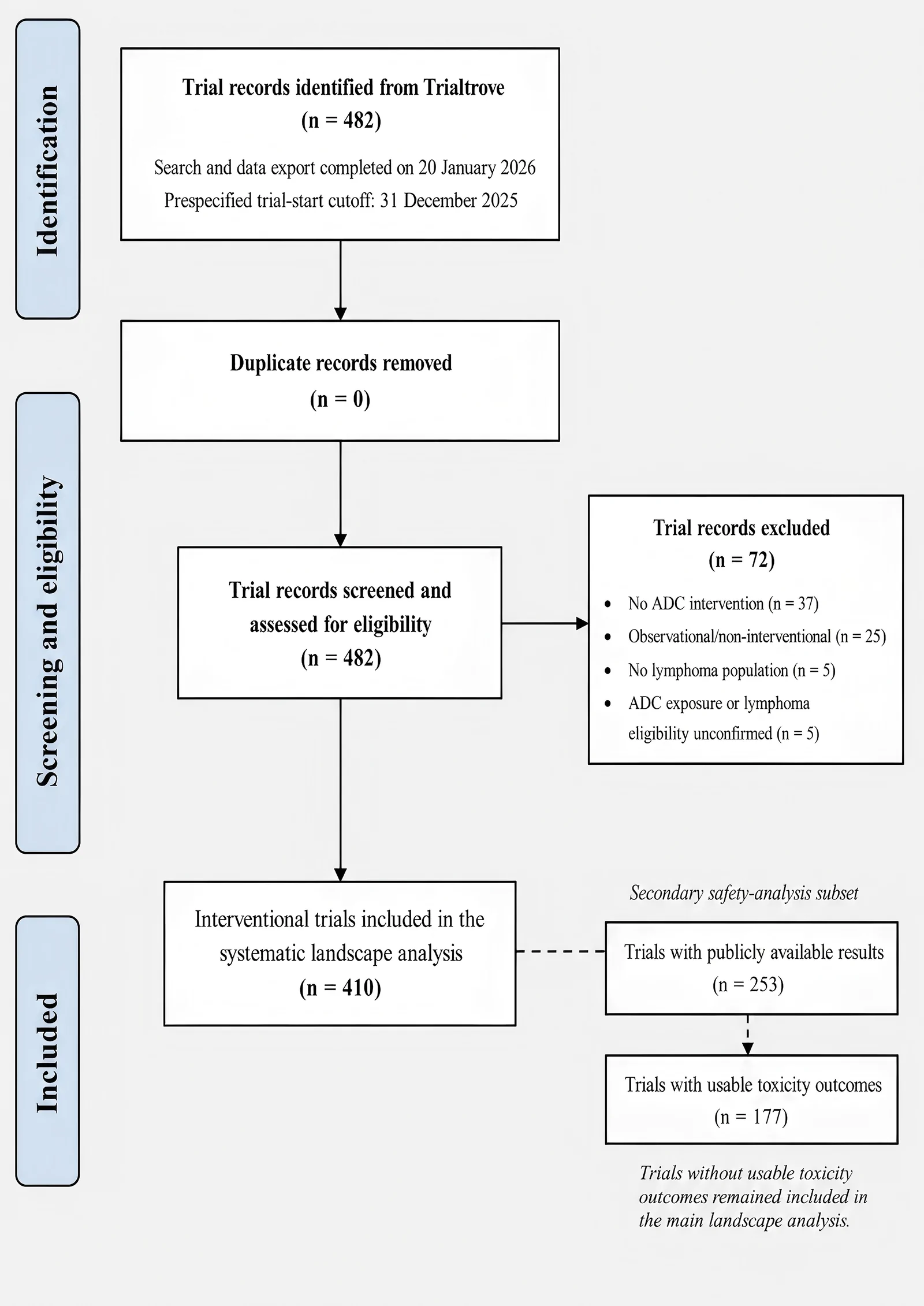
PRISMA-based flow diagram of trial identification, screening, eligibility assessment, and inclusion. Trial records were retrieved from the Trialtrove database using prespecified filters for antibody–drug conjugates, Hodgkin or non-Hodgkin lymphoma, trial start date, and clinical phase. The search and data export were completed on 20 January 2026, with a prespecified trial-start cutoff of 31 December 2025. A total of 482 records were identified, and no duplicate records were found after comparison of trial identifiers, sponsor protocol numbers, and study titles. After screening and eligibility assessment, 72 records were excluded because they did not evaluate an ADC (n = 37), were observational or otherwise non-interventional (n = 25), did not include patients with lymphoma (n = 5), or lacked sufficient information to confirm the ADC intervention or lymphoma eligibility criteria (n = 5). The final systematic landscape analysis included 410 interventional trials. Among these, 253 had publicly available results, and 177 reported at least one usable toxicity outcome and were included in the secondary safety analysis. Trials without usable toxicity outcomes remained included in the main landscape analysis. The complete Trialtrove retrieval strategy is provided in Section 2.1.

The search identified 482 records, of which 72 were excluded: 37 did not evaluate an ADC, 25 were observational, 5 did not include patients with lymphoma, and 5 lacked sufficient information to confirm the ADC intervention or lymphoma eligibility. The final dataset comprised 410 interventional trials (**Figure 1**; **Supplementary Table 1**).

**Figure 2:**
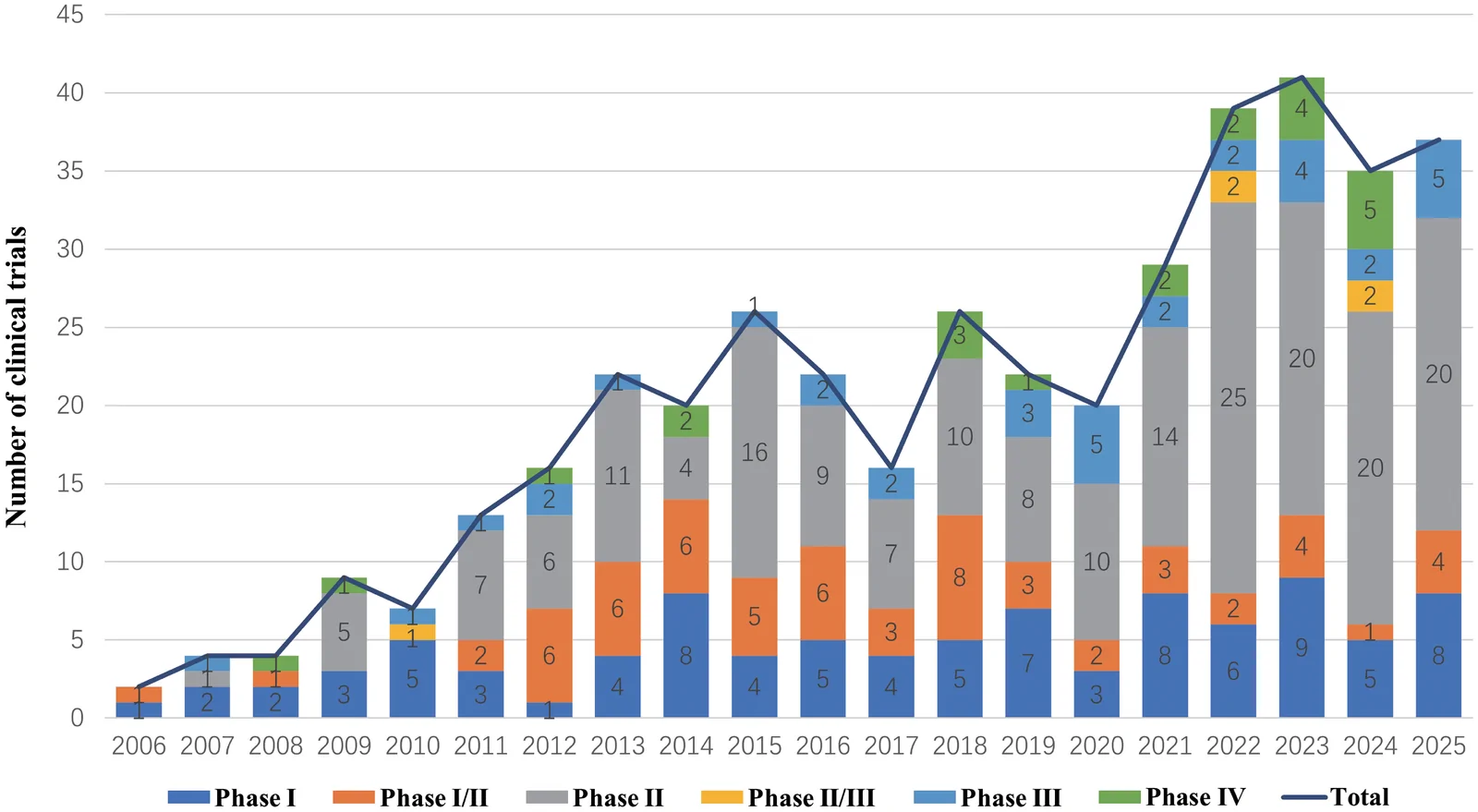
Temporal distribution of lymphoma ADC trials by start year. Counts are based on individual interventional trials (n = 410), with each trial counted once. Start year was defined using the actual start date when available and the anticipated start date for planned trials without an actual start date. No eligible Phase III/IV trials were identified; therefore, this category is not displayed.

Annual trial initiation increased from 2 trials in 2006 to 37 in 2025 and peaked at 41 in 2023. Overall, 201 trials (49.0%) began between 2020 and 2025, compared with 26 during 2006–2010. Phase II was the largest category, with 193 trials (47.1%), followed by Phase I with 93 (22.7%) and Phase I/II with 63 (15.4%). Late-stage development included 34 Phase III trials (8.3%), 22 Phase IV trials (5.4%), and 5 Phase II/III trials (1.2%). No eligible Phase III/IV trial was identified (**Figure 2**).

**Figure 3:**
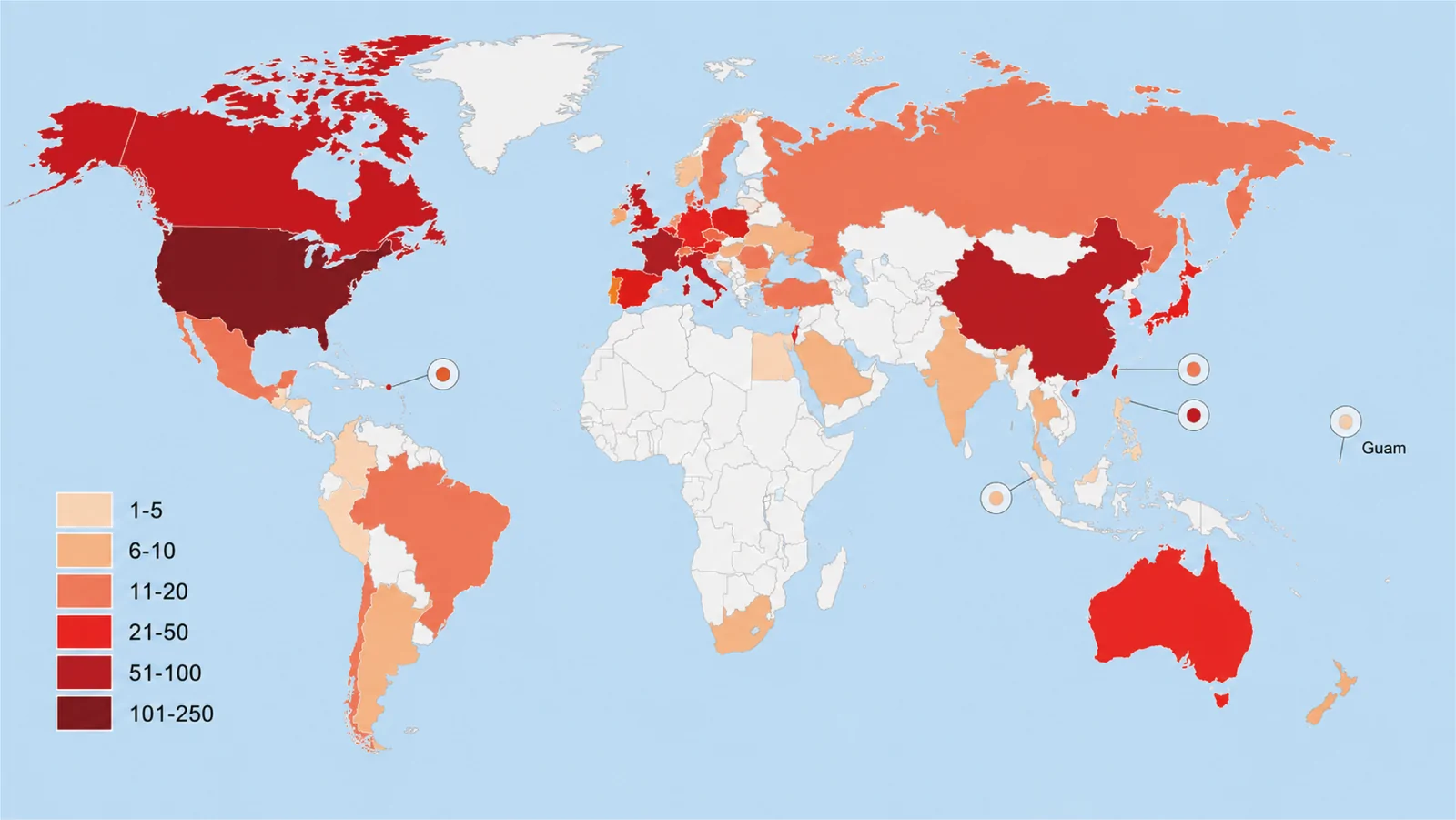
Global distribution of ADC clinical trials in lymphoma treatment. The map shows the geographic distribution of trial activity at the country level. Counts are based on trial–country records rather than unique trials. Each multinational trial contributed one record to each participating country; therefore, the summed country-level counts could exceed the 410 included interventional trials. Countries were shaded according to the number of associated trial–country records, grouped into the predefined categories shown in the legend.

Geographic participation was broad. Of the 410 trials, 311 (75.9%) were conducted within a single continent, most commonly North America (161, 39.3%), Asia (89, 21.7%), or Europe (56, 13.7%). The remaining 99 trials (24.1%) involved multiple continents. Europe–North America was the most frequent combination (33, 8.0%), followed by Asia–Europe–North America (17, 4.1%) and Asia–Europe–North America–Oceania (11, 2.7%). When all geographic participation was counted, North America was represented in 251 trials (61.2%), Europe in 148 (36.1%), and Asia in 143 (34.9%); Oceania, South America, and Africa were represented in 37, 18, and 9 trials, respectively (**Figure 3**).

**Figure 4:**
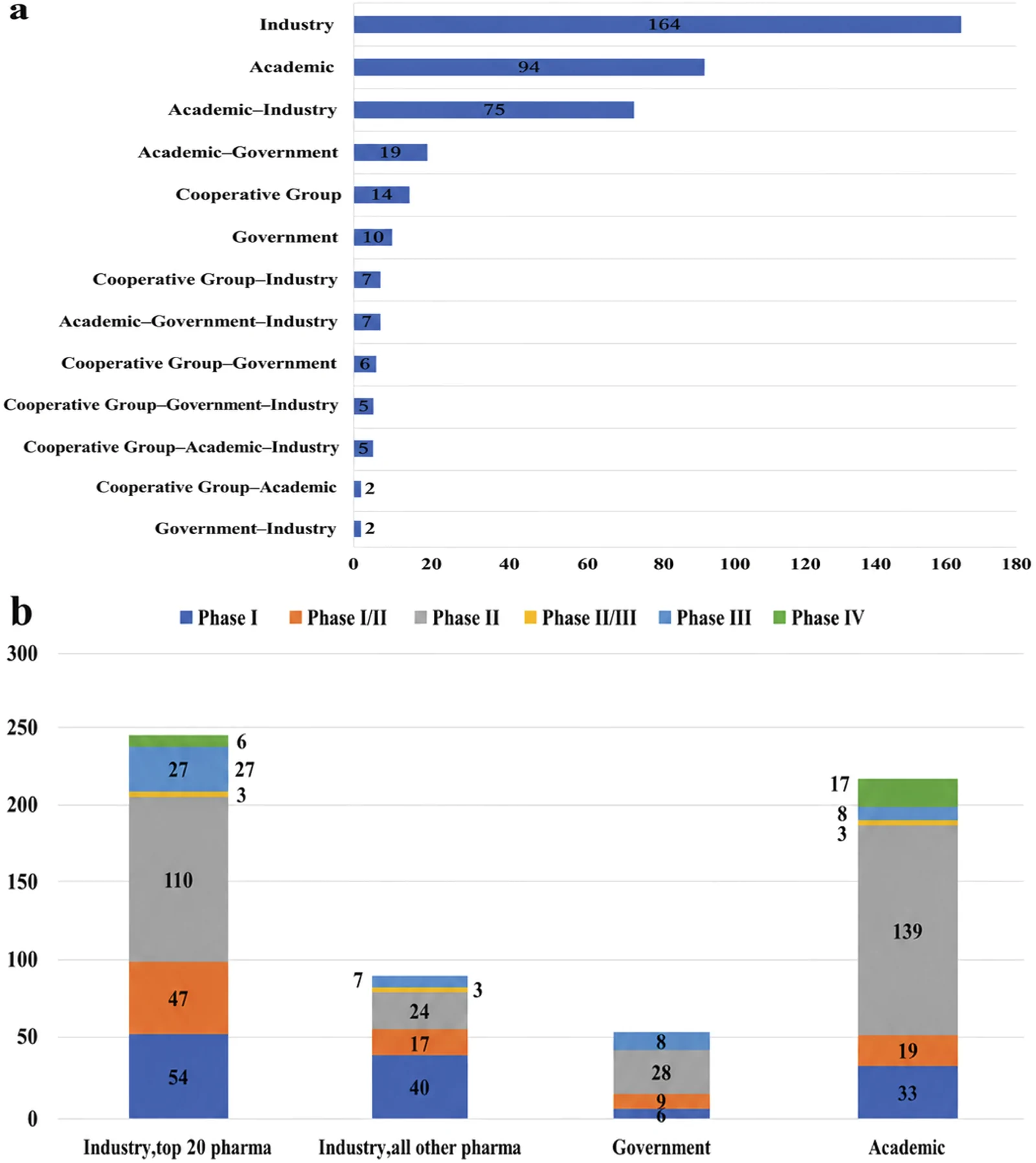
Funding patterns and sponsor involvement in lymphoma ADC trials. **(A)** Distribution of mutually exclusive trial-level funding categories. Each of the 410 trials was assigned to one funding category, and percentages were calculated using all included trials as the denominator. **(B)** Phase distribution of sponsor involvement by sponsor type at the sponsor–trial record level. Sponsor categories were not mutually exclusive because one trial could involve more than one sponsor type. A trial contributed one sponsor–trial record for each applicable sponsor category; therefore, totals could exceed the number of unique trials.

At the trial level, industry-only funding was most frequent (164, 40.0%), followed by academic-only funding (94, 22.9%) and academic–industry funding (75, 18.3%). Academic–government, cooperative-group, and government-only funding supported 19 (4.6%), 14 (3.4%), and 10 trials (2.4%), respectively, while other mixed arrangements accounted for 34 (8.3%) (**Figure 4A**).

Sponsor–trial categories were not mutually exclusive. Top-20 pharmaceutical companies were involved in 247 records, including 110 Phase II and 27 Phase III records. Other pharmaceutical companies contributed 91 records and were concentrated in Phase I (40, 44.0%) and Phase I/II or Phase II development. Academic institutions contributed 219 records but only 8 Phase III records, whereas 28 of 51 government-sponsored records were Phase II. Later-stage involvement was therefore more evident among top-20 pharmaceutical companies, while academic and government participation remained concentrated in Phase II development (**Figure 4B**).

### 3.2 Lymphoma subtype heterogeneity in ADC trial development

Subtype analyses used trial–subtype pairs because several trials enrolled more than one lymphoma subtype. The 410 trials generated 837 trial–subtype pairs. DLBCL was the largest subtype category, with 213 pairs (25.4%), followed by cHL with 135 (16.1%), FL with 90 (10.8%), PTCL with 70 (8.4%), and MCL with 67 (8.0%). Together, these five categories accounted for 575 pairs (68.7%). NLPHL, extranodal MZL/MALT, LBL, CTCL, CLL/SLL, WM/LPL, ALCL, HGBL, and PMBCL were less frequently represented.

**Figure 5:**
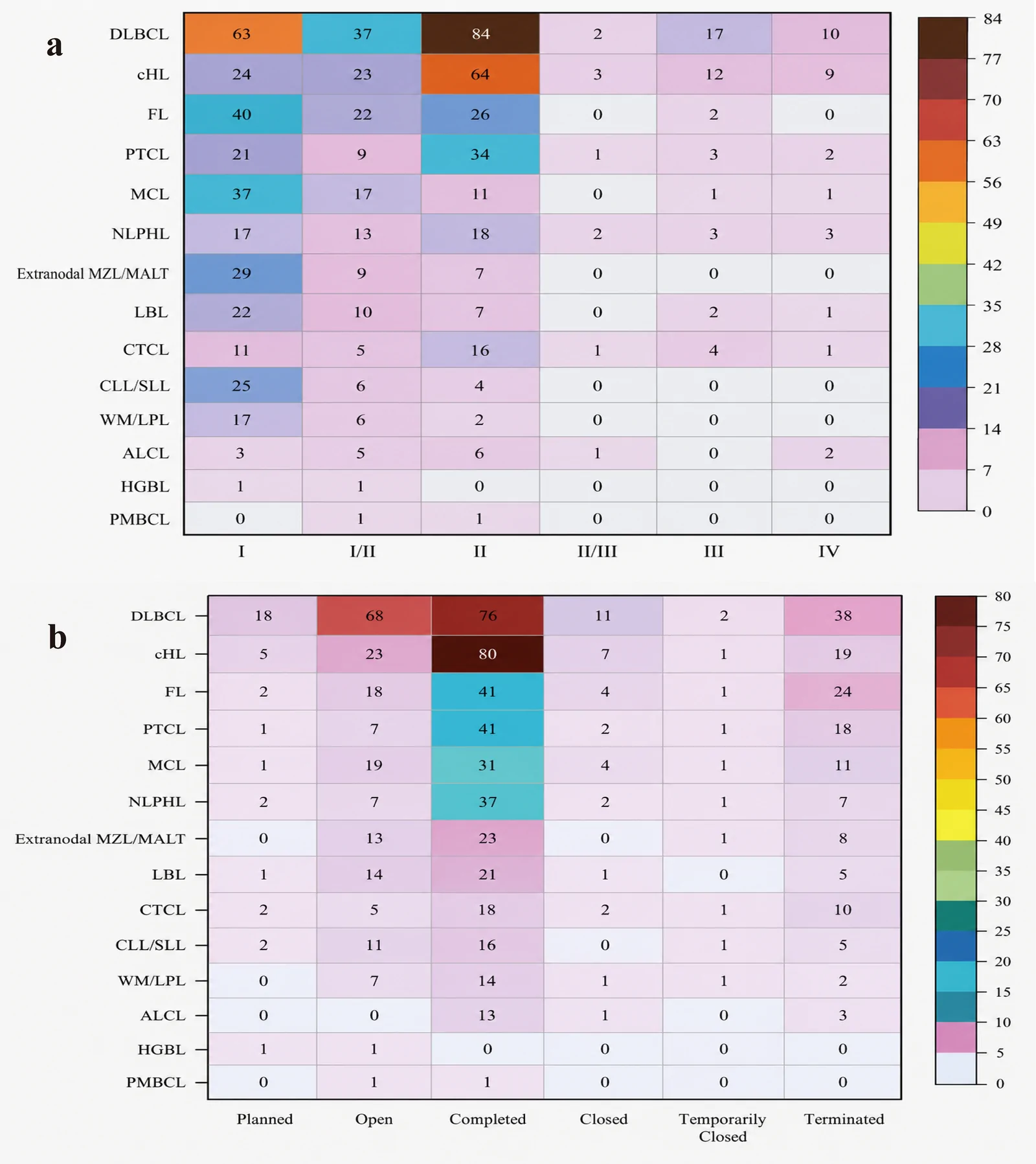
**(A)** Lymphoma subtype distribution by trial phase **(B)** Lymphoma subtype distribution by trial status. Counts are based on trial–subtype pairs. Each trial contributed one record for each eligible lymphoma subtype, and the trial-level phase or status classification was assigned to every corresponding subtype pair. Because some trials enrolled more than one lymphoma subtype, totals exceeded the 410 unique trials.

Phase distribution differed by subtype. DLBCL had the broadest profile, including 84 Phase II, 17 Phase III, and 10 Phase IV pairs. cHL also showed comparatively mature development, with 64 Phase II, 12 Phase III, and 9 Phase IV pairs. By contrast, Phase I accounted for 44.4% of FL pairs, 55.2% of MCL pairs, 64.4% of extranodal MZL/MALT pairs, 71.4% of CLL/SLL pairs, and 68.0% of WM/LPL pairs. Overall, only 83 of 837 trial–subtype pairs (9.9%) were Phase II/III, Phase III, or Phase IV, and late-phase activity was concentrated mainly in DLBCL and cHL (**Figure 5A**).

Trial status varied across the same disease groups. Completed, open, and terminated studies accounted for 412 (49.2%), 194 (23.2%), and 150 (17.9%) trial–subtype pairs, respectively. DLBCL had the largest numbers of both open and terminated pairs, while cHL had the largest number of completed pairs. FL and PTCL included 24 and 18 terminated pairs, respectively. Completed status was treated as an operational outcome rather than evidence of clinical benefit (**Figure 5B**).

### 3.3 Trial phase, study design, and primary endpoint patterns

**Figure 6:**
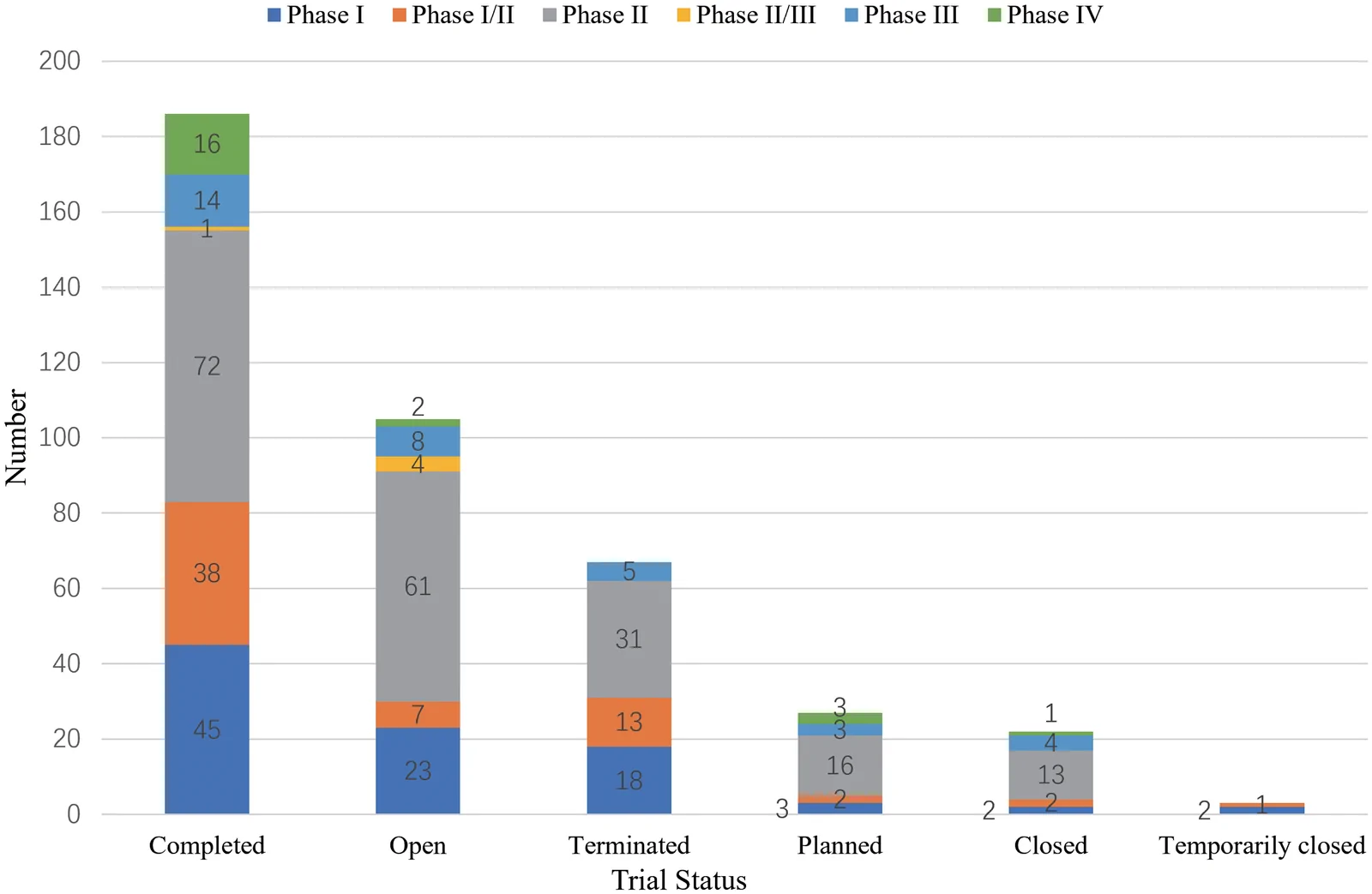
Trial phase and trial status distribution of lymphoma ADC trials. Counts are based on individual interventional trials (n = 410). Each trial was assigned to one clinical phase and one trial status category and therefore contributed once to the phase-by-status distribution.

At the trial level, Phase II accounted for 72 of 186 completed trials, 61 of 105 open trials, and 31 of 67 terminated trials. Phase I and Phase I/II studies also contributed substantially to completed and terminated categories, whereas late-phase trials remained uncommon across trial statuses (**Figure 6**).

Single-arm designs predominated, accounting for 253 trials (61.7%). Multi-arm designs were used in 130 (31.7%), and arm structure was unclear in 27 (6.6%). Only 76 trials (18.5%) were randomized; 310 (75.6%) were non-randomized, and randomization status was unavailable for 24. Randomized designs became more common after 2010. They accounted for 2 of 26 trials (7.7%) initiated during 2006–2010, 18 of 97 (18.6%) during 2011–2015, 21 of 106 (19.8%) during 2016–2020, and 35 of 181 (19.3%) during 2021–2025. Thus, the use of randomization increased compared with the earliest period but remained close to one fifth of trials after 2010, without a sustained upward trend.

Primary endpoint categories were non-mutually exclusive. Safety-related endpoints appeared in 205 trials (50.0%), ORR or another response-rate endpoint in 173 (42.2%), and CR/CMR in 153 (37.3%). DLT and MTD were used in 100 (24.4%) and 90 trials (22.0%), respectively. PFS appeared in 78 trials (19.0%) and OS in 26 (6.3%). DOR, PK, immunogenicity, RP2D/RDE, MRD, and biomarker or correlative endpoints were uncommon; MRD and biomarker-related endpoints each appeared in one trial.

**Figure 7:**
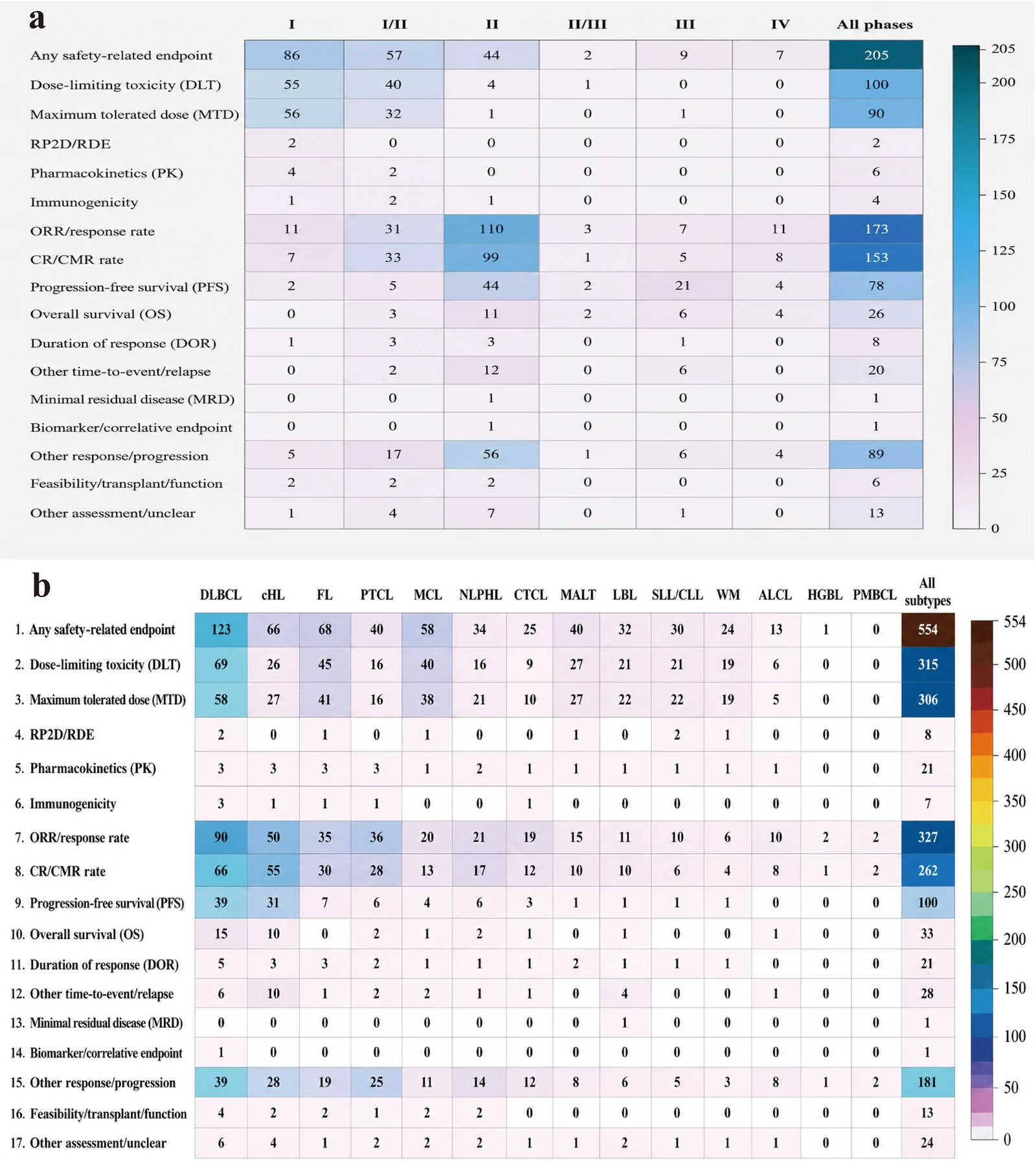
**(A)** Primary endpoint categories across trial phases. Counts are based on trial–endpoint category records. Each trial was counted no more than once within a given endpoint category but could contribute to multiple categories when more than one primary endpoint category was reported. “Any safety-related endpoint” was a composite category that included DLT and MTD, which were also displayed separately to characterize specific dose-finding endpoint use. **(B)** Primary endpoint categories across lymphoma subtypes Counts are based on trial–subtype–endpoint category records. A trial could contribute more than one record when it enrolled multiple lymphoma subtypes, reported multiple primary endpoint categories, or both. These counts therefore represent analytical records rather than unique trials.

The composite safety category included DLT and MTD, which were also displayed separately. Among Phase I trials, 86 included a safety-related endpoint, including 55 with DLT and 56 with MTD; only 11 included ORR and 7 included CR/CMR. Phase I/II studies had a mixed profile, with 57 safety-related, 31 ORR, and 33 CR/CMR endpoints. Phase II trials were predominantly response-oriented: ORR appeared in 110, CR/CMR in 99, and PFS in 44 trials. Among the five Phase II/III trials, ORR appeared in three, whereas safety, PFS, and OS each appeared in two. In Phase III, PFS was the leading endpoint, appearing in 21 of 34 trials (61.8%); safety, ORR, and OS appeared in 9, 7, and 6 trials, respectively. Phase IV studies remained mainly response-oriented, with ORR in 11 of 22 trials, CR/CMR in 8, safety endpoints in 7, and PFS and OS in 4 each. No Phase IV trial used DLT, MTD, RP2D/RDE, PK, immunogenicity, DOR, MRD, or biomarker endpoints as a primary endpoint category (**Figure 7A**).

Subtype-stratified analysis used trial–subtype–endpoint category records. Safety-related endpoints generated 554 records, followed by ORR or response rate (327), DLT (315), MTD (306), CR/CMR (262), and PFS (100); OS generated 33 records. DLBCL contributed the largest number of safety (123), ORR (90), CR/CMR (66), PFS (39), and OS records (15). cHL contributed 66, 50, 55, 31, and 10 records in the same categories. Other subtypes remained more dependent on safety- and response-based endpoints, with fewer time-to-event or biomarker-related measures (**Figure 7B**). Selected landmark and contemporary studies are summarized in **Table 1** and **Supplementary Table 2**.

**Table 1:** Selected landmark and contemporary clinical trials illustrating the development of antibody–drug conjugates in lymphoma.

Start year	Trial/identifiers	ADC platform	Population and treatment setting	Phase, status, and design	Regimen/comparator	Sample size	Primary endpoint	Key efficacy findings	Key safety findings	Developmental or regulatory relevance
2009	SG035-0003 Trialtrove ID 128828 NCT00848926	Brentuximab vedotin CD30 | MMAE | cleavable	R/R cHL after autologous stem-cell transplantation; heavily pretreated	Phase II; completed Single-arm, non-randomized	Brentuximab vedotin monotherapy No comparator	102	ORR by independent review	ORR 75%; CR 34%; median PFS 5.6 months; median DOR among patients with CR 20.5 months	Grade ≥3 neutropenia 20%, peripheral sensory neuropathy 8%, thrombocytopenia 8%, and anemia 6%; approximately 20% discontinued because of adverse events	Pivotal single-arm study supporting the initial regulatory development of brentuximab vedotin in R/R cHL
2010	AETHERA Trialtrove ID 128106 NCT01100502	Brentuximab vedotin CD30 | MMAE | cleavable	cHL at high risk of relapse or progression after autologous HSCT	Phase III; completed Randomized, double-blind, placebo-controlled	Brentuximab vedotin + best supportive care vs placebo + best supportive care	329	PFS by independent review	Median PFS 42.9 vs 24.1 months; HR 0.57 (95% CI 0.40–0.81); p=0.0013	Peripheral sensory neuropathy 56% (grade 3: 10%); peripheral motor neuropathy 23%; grade 3/4 neutropenia 29%; adverse-event-related discontinuation approximately 33%	Established post-transplant consolidation as a clinically distinct ADC treatment setting
2012	ECHELON-1 Trialtrove ID 151814 NCT01712490	Brentuximab vedotin CD30 | MMAE | cleavable	Previously untreated stage III or IV cHL	Phase III; completed Randomized, open-label	A+AVD vs ABVD	1334	Modified PFS	Two-year modified PFS 82.1% vs 77.2%; HR 0.77 (95% CI 0.60–0.98). Six-year OS 93.9% vs 89.4%; HR 0.59 (95% CI 0.40–0.88)	Peripheral neuropathy 67% vs 43%; grade ≥3 neutropenia and febrile neutropenia were more frequent with A+AVD, supporting primary G-CSF prophylaxis	Moved a CD30-directed ADC into frontline treatment for advanced cHL and subsequently demonstrated an OS benefit
2013	ECHELON-2 Trialtrove ID 151812 NCT01777152	Brentuximab vedotin CD30 | MMAE | cleavable	Previously untreated CD30-positive PTCL, predominantly systemic ALCL	Phase III; completed Randomized, double-blind, active-controlled	A+CHP vs CHOP	452	PFS by independent review	Median PFS 48.2 vs 20.8 months; HR 0.71 (95% CI 0.54–0.93). CR 68% vs 56%; ORR 83% vs 72%; OS HR 0.66 (95% CI 0.46–0.95)	Peripheral neuropathy 52% with A+CHP; major grade ≥3 toxicities were hematologic, including neutropenia and febrile neutropenia	Provided randomized phase III evidence for frontline ADC-containing therapy in CD30-positive PTCL/sALCL
2017	POLARIX Trialtrove ID 308672 NCT03274492	Polatuzumab vedotin CD79b | MMAE | cleavable	Previously untreated intermediate/high-risk DLBCL (IPI 2–5)	Phase III; completed Randomized, double-blind, placebo-controlled	Pola-R-CHP vs R-CHOP	879	PFS	Two-year PFS 76.7% vs 70.2%; HR 0.73 (95% CI 0.57–0.95); p=0.02. Two-year OS 88.7% vs 88.6%; HR 0.94	Grade 3/4 neutropenia 28.3% vs 30.8%, febrile neutropenia 13.8% vs 8.0%, and anemia 12.0% vs 8.4%; overall safety profiles were broadly similar	Supported frontline polatuzumab-R-CHP for previously untreated DLBCL/HGBL with IPI ≥2
2018	LOTIS-2 Trialtrove ID 328309 NCT03589469	Loncastuximab tesirine CD19 | PBD | cleavable	R/R DLBCL after at least two prior systemic regimens	Phase II; completed Single-arm, non-randomized	Loncastuximab tesirine monotherapy No comparator	145	ORR by independent review	ORR 48.3% (95% CI 39.9–56.7); CR 24.1–24.8%; median DOR 13.4 months in long-term follow-up	Most common grade ≥3 events: neutropenia 26%, thrombocytopenia 18%, increased GGT 17%, and anemia 10%; serious adverse events occurred in 39%	Single-arm registrational evidence supporting accelerated approval after at least two prior lines of systemic therapy
2020	ECHELON-3 Trialtrove ID 375436 NCT04404283	Brentuximab vedotin CD30 | MMAE | cleavable	R/R LBCL after at least two prior lines; ineligible for autologous HSCT or CAR-T-cell therapy	Phase III; completed Randomized, double-blind, placebo-controlled	Brentuximab vedotin + lenalidomide + rituximab vs placebo + lenalidomide + rituximab	230	OS	Median OS 13.8 vs 8.5 months; HR 0.63 (95% CI 0.45–0.89); p=0.0085. Median PFS 4.2 vs 2.6 months; ORR 64.3% vs 41.5%	Common adverse reactions included fatigue, diarrhea, peripheral neuropathy, rash, and pneumonia; peripheral neuropathy occurred or worsened in 27%, causing dose reduction in 6% and discontinuation in 4.5%	Supported a 2025 approval of the triplet for R/R LBCL in patients ineligible for autologous HSCT or CAR-T-cell therapy
2025	SHR-A1912 phase III Trialtrove ID 569811 NCT06929624	SHR-A1912 CD79b | TOP1i | cleavable	R/R DLBCL	Phase III; open Randomized, open-label	SHR-A1912 + R-GemOx vs R-GemOx	280	OS and response rate	No phase III efficacy results available; recruitment/development was ongoing at the database cutoff	No phase III safety results available	Contemporary late-phase program illustrating expansion from MMAE-based platforms to a CD79b-directed topoisomerase I inhibitor ADC

Selection was illustrative rather than exhaustive. Efficacy and safety values were reproduced from the cited primary publications, long-term follow-up reports, regulatory documents, or registry records and were not pooled. For LOTIS-2, the updated final CR rate (24.8%) and median DOR (13.4 months) are shown; the primary analysis reported a CR rate of 24.1%. No phase III efficacy or safety results were available for SHR-A1912 at the database cutoff (20 January 2026).

A+AVD, brentuximab vedotin plus doxorubicin, vinblastine, and dacarbazine; ABVD, doxorubicin, bleomycin, vinblastine, and dacarbazine; A+CHP, brentuximab vedotin plus cyclophosphamide, doxorubicin, and prednisone; ADC, antibody–drug conjugate; AE, adverse event; ALCL, anaplastic large cell lymphoma; CAR T, chimeric antigen receptor T-cell; cHL, classical Hodgkin lymphoma; CHOP, cyclophosphamide, doxorubicin, vincristine, and prednisone; CI, confidence interval; CR, complete response; CRR, complete response rate; DLBCL, diffuse large B-cell lymphoma; DOR, duration of response; G-CSF, granulocyte colony-stimulating factor; GGT, γ-glutamyl transferase; HGBL, high-grade B-cell lymphoma; HR, hazard ratio; HSCT, hematopoietic stem-cell transplantation; IPI, International Prognostic Index; LBCL, large B-cell lymphoma; MMAE, monomethyl auristatin E; ORR, objective response rate; OS, overall survival; PBD, pyrrolobenzodiazepine; PFS, progression-free survival; PTCL, peripheral T-cell lymphoma; R-GemOx, rituximab, gemcitabine, and oxaliplatin; R/R, relapsed or refractory; TEAE, treatment-emergent adverse event.

### 3.4 Target dynamics, program-level developmental maturity, and inactive trial patterns

**Figure 8:**
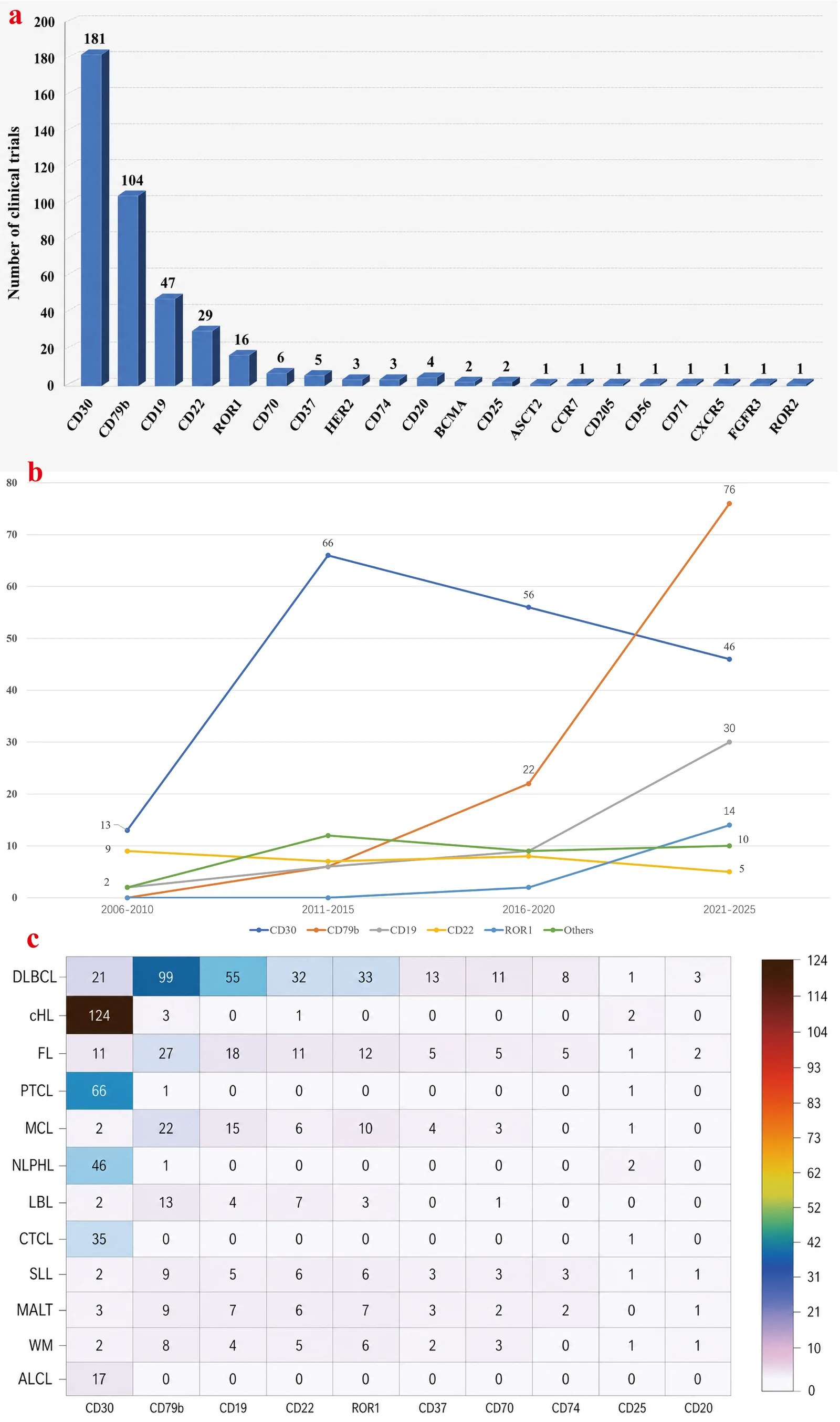
Target distribution and target development dynamics in lymphoma ADC trials. **(A)** Overall target distribution. Counts are based on target–trial records because some trials involved more than one ADC target. **(B)** Period-based trends for the top five targets and other targets. Counts are based on target–trial records stratified by trial-start period. A trial evaluating more than one confirmed ADC target could contribute more than one record within the corresponding period. “Other targets” comprised all confirmed ADC targets outside the five most frequent categories. **(C)** Distribution of the top 10 ADC targets across lymphoma subtypes. Counts are based on trial–subtype–target records. A trial could contribute more than one record when it enrolled multiple lymphoma subtypes, evaluated more than one confirmed ADC target, or both. Counts therefore represent analytical records rather than unique trials.

Target analysis identified 181 CD30 target–trial records, 104 CD79b records, 47 CD19 records, 29 CD22 records, and 16 ROR1 records. Other targets, including CD70, CD37, CD20, CD74, CD25, BCMA, and HER2, were less frequent (**Figure 8A**).

Target use changed over time. CD30 dominated the earlier landscape, increasing from 13 records in 2006–2010 to 66 in 2011–2015, before declining to 56 in 2016–2020 and 46 in 2021–2025. CD79b was absent in 2006–2010 but increased to 22 records in 2016–2020 and 77 in 2021–2025. CD19 rose from 2 to 31 records across the first and most recent periods, while ROR1 increased from 2 records in 2016–2020 to 14 in 2021–2025. CD22 changed less substantially (**Figure 8B**).

Development was also examined at the unique ADC program level. Each program was counted once according to the highest phase reached in lymphoma trials. Fifty unique ADC programs were identified. Thirty-five (70.0%) reached no phase beyond Phase I or Phase I/II, eight (16.0%) reached Phase II, four (8.0%) reached Phase II/III or Phase III, and three (6.0%) reached Phase IV. These categories represent the highest phase attained rather than phase-transition rates.

Trial–subtype–target records reflected established lymphoma biology. CD30 activity was concentrated in cHL and mature T-cell lymphomas, including 124 cHL, 66 PTCL, 35 CTCL, and 17 ALCL records. Most NLPHL records arose from mixed-subtype trials and should be interpreted as eligibility rather than evidence of uniform CD30 expression. CD79b was concentrated in DLBCL, which contributed 99 records. CD19 and CD22 had broader B-cell distributions, including 55 and 32 DLBCL records, respectively. ROR1, CD37, CD70, and CD74 remained less frequently studied (**Figure 8C**).

**Figure 9:**
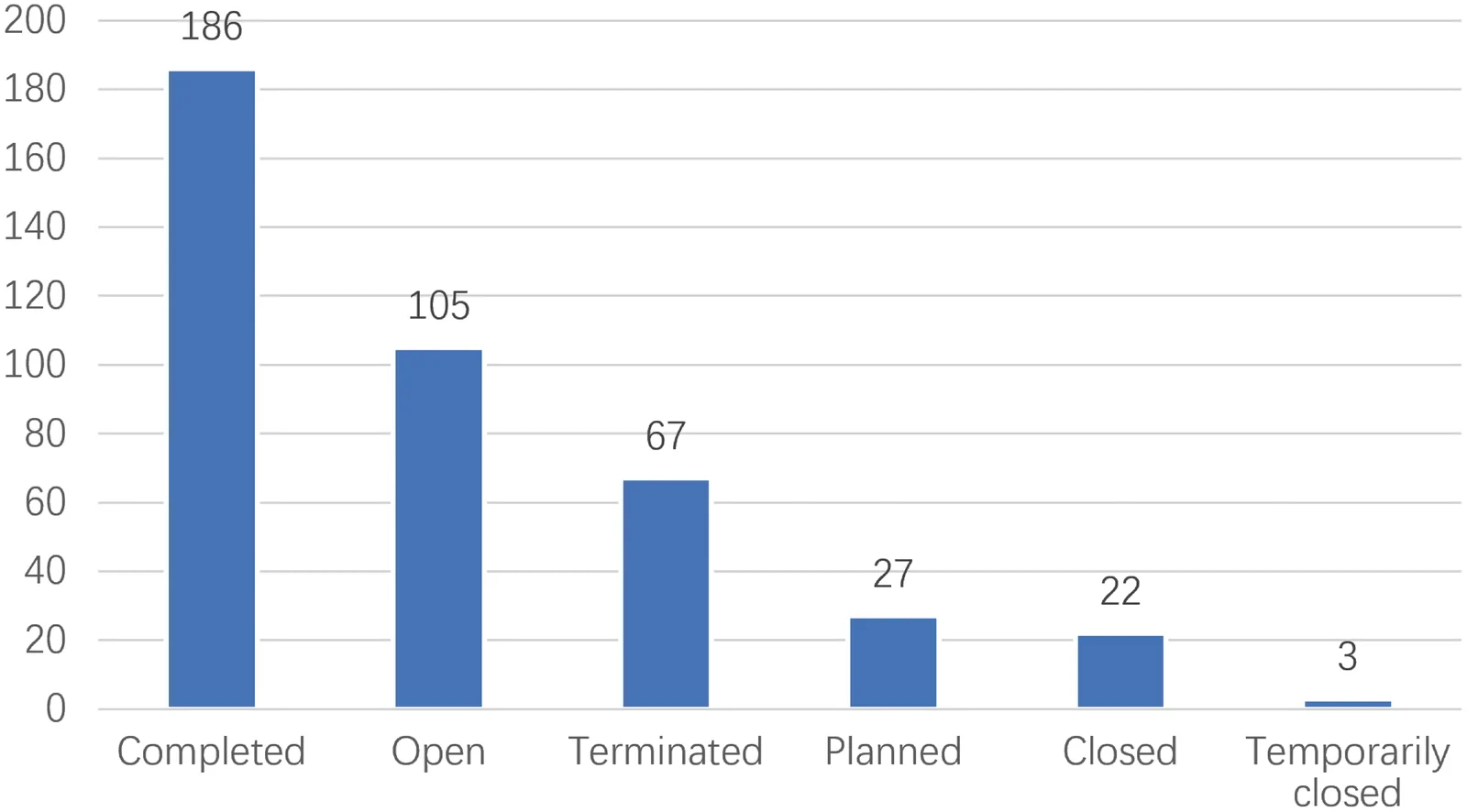
Distribution of clinical trial status. Counts are based on individual interventional trials (n = 410). Each trial was assigned to one mutually exclusive status category according to its standardized status at the time of data export on 20 January 2026.

Overall, 186 trials were completed, 105 were open, 67 were terminated, 27 were planned, 22 were closed, and 3 were temporarily closed (**Figure 9**). The 67 terminated, 22 closed, and 3 temporarily closed trials formed 92 inactive-status records.

Recruitment or feasibility problems were reported in 30 inactive records (32.6%), no specific reason was available for 28 (30.4%), and sponsor strategy or pipeline reprioritization accounted for 15 (16.3%). Insufficient efficacy and safety or adverse effects were each reported in five records; four had no further development reported, three had administrative or other reasons, and two had funding-related reasons.

Target analysis of the 92 inactive-status trials identified CD30 in 39 records (42.4%), CD79b in 17 (18.5%), and CD19 in 16 (17.4%). CD22 and ROR1 each appeared in four. CD37 appeared in three, while CD70 and CD74 each appeared twice. Among CD30-directed inactive records, recruitment or feasibility problems were reported in 17, no reason was available for 11, and sponsor strategy or reprioritization explained 5. For CD79b, eight records lacked a reported reason, five involved recruitment or feasibility, and two involved safety. CD19 showed a different distribution: six records were linked to sponsor strategy, four to recruitment, and three had no reported reason. Because commonly studied targets had more opportunities to generate inactive records, these counts do not represent target-specific failure rates.

The 92 inactive trials involved 23 ADCs. Brentuximab vedotin appeared in 37 records (40.2%), polatuzumab vedotin in 17 (18.5%), and loncastuximab tesirine in 11 (12.0%). Among the 67 terminated trials, the corresponding counts were 29, 9, and 9. Their high frequencies largely reflected greater trial volume and longer development histories rather than comparative failure.

Cleavable linkers were used in 83 inactive trials (90.2%), non-cleavable linkers in 8, and an unknown linker in 1. Recruitment, missing explanations, and sponsor strategy were the leading reasons among cleavable-linker trials. The marked imbalance between linker groups precluded meaningful comparison of linker-specific inactivity.

Overall, this analysis shows that target development in lymphoma ADC trials is highly concentrated but not static. CD30 remains central in cHL and CD30-positive T-cell lymphoma settings. CD79b, CD19, and CD22 dominate B-cell lymphoma ADC development, especially in DLBCL and related B-cell subtypes. Newer targets, such as ROR1, CD70, CD37, and CD74, are being explored, but they remain less frequent than the established target group. Most reported reasons were linked to recruitment, feasibility, sponsor strategy, or missing public explanation. This finding supports a cautious interpretation of trial status and shows why trial completion, termination, or closure should not be used alone as a marker of clinical value.

### 3.5 Payload and linker platform patterns across lymphoma subtypes

**Figure 10:**
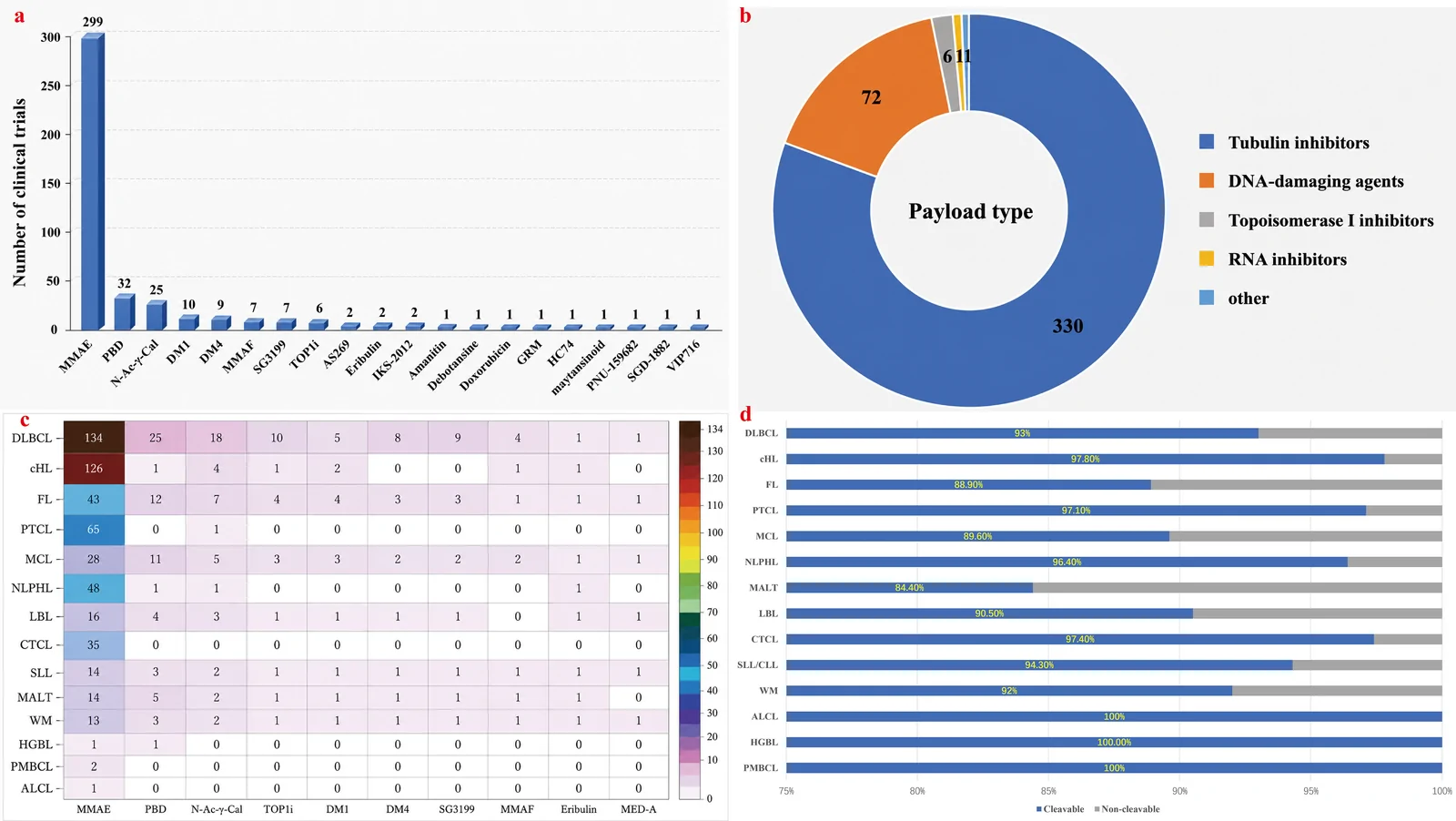
Payload and linker platform patterns across lymphoma subtypes. **(A)** Overall distribution of individual payloads in lymphoma ADC trials. Counts are based on the payload conjugated to the primary ADC assigned to each trial. Each of the 410 trials contributed no more than one payload assignment to the overall distribution. **(B)** Distribution of payload mechanism classes in lymphoma ADC trials. Counts are based on the mechanism class of the payload conjugated to the primary ADC assigned to each trial. Each trial contributed no more than one payload-class assignment. **(C)** Distribution of the top 10 payloads across lymphoma subtypes. Counts are based on trial–subtype–payload records. The payload was derived from the primary ADC assigned to each trial. A trial enrolling multiple lymphoma subtypes contributed one record for each subtype but no more than one payload assignment within each trial–subtype pair. Totals could therefore exceed the number of unique trials. **(D)** Linker type distribution across lymphoma subtypes. Counts are based on trial–subtype–linker records. Linker type was derived from the primary ADC assigned to each trial. A trial enrolling multiple lymphoma subtypes contributed one linker record for each subtype. Percentages were calculated within each lymphoma subtype. Percentages were calculated among trial–subtype–linker records with a known linker classification; records with an undisclosed or unclear linker type were excluded from the percentage denominator.

Payload analyses were conducted at the payload–trial record level. Tubulin inhibitors were the dominant payload class, with 330 records (80.5%). DNA-damaging agents accounted for 72 records (17.6%). Topoisomerase I inhibitors were recorded in 6 records (1.5%). RNA inhibitors and other payloads were each recorded in 1 record (0.2%). At the individual payload level, MMAE was the most frequent payload, with 299 records (72.9%). PBD and N-Ac-γ-Cal followed, with 32 (7.8%) and 25 (6.1%) records, respectively. Other payloads were less common, including DM1, DM4, MMAF, SG3199, TOP1i, AS269, eribulin, IKS-2012, amanitin, debotansine, doxorubicin, GRM, HC74, maytansinoid, MED-A, PNU-159682, SGD-1882, and VIP716. These results show that lymphoma ADC development is still heavily based on a small number of cytotoxic payload platforms, especially MMAE-based tubulin inhibition (**Figure 10A**).

At the mechanism level, tubulin inhibitors dominated, driven mainly by MMAE and smaller contributions from maytansinoids, MMAF, eribulin, and related agents. DNA-damaging payloads were the second major class, whereas topoisomerase I inhibitors, RNA-directed agents, and other mechanisms remained uncommon (**Figure 10B**).

Subtype–payload analysis used non-mutually exclusive subtype–payload records. MMAE predominated across nearly all major lymphoma subtypes. DLBCL and cHL contributed 134 and 126 trial–subtype–payload records, respectively. DLBCL showed the widest payload diversity, including PBD, N-Ac-γ-Cal, topoisomerase I inhibitors, DM1, DM4, SG3199, MMAF, eribulin, and MED-A. FL and MCL also showed some diversity, whereas cHL and mature T-cell lymphomas were more closely associated with MMAE-based platforms (**Figure 10C**).

Cleavable linkers predominated in every major subtype, accounting for 93.0% of DLBCL, 97.8% of cHL, 88.9% of FL, 97.1% of PTCL, 89.6% of MCL, 96.4% of NLPHL, 84.4% of extranodal MZL/MALT, and 90.5% of LBL trial–subtype–linker records. Non-cleavable linkers were present in several subtypes, but they did not dominate any subtype group. They were relatively more visible in MALT, FL, MCL, LBL, WM, and DLBCL, but their overall contribution remained limited (**Figure 10D**).

MMAE-based tubulin inhibition and cleavable linkers therefore remained the main technical platform across lymphoma ADC trials. Payload exploration was broader in DLBCL, FL, and MCL than in cHL or mature T-cell lymphoma.

### 3.6 Combination strategies and immunotherapy-related trial designs

All 410 trials included at least one ADC. Of these, 232 (56.6%) did not include a non-ADC partner, while 178 (43.4%) used at least one additional therapy. Partner categories were not mutually exclusive because a single trial could include more than one class.

Chemotherapy or corticosteroids were used in 82 trials (20.0%). Other monoclonal antibodies or immunotherapy partners, excluding checkpoint inhibitors and bispecific antibodies or T-cell engagers, appeared in 62 trials (15.1%). Targeted small molecules or immunomodulatory agents were used in 61 (14.9%). Immune checkpoint inhibitors appeared in 35 trials (8.5%), and bispecific antibodies or T-cell engagers in 31 (7.6%). Ten trials involved cellular therapy or transplant-related strategies. Eight used supportive, other, or unidentified agents.

At least one immune-based partner was present in 123 trials (30.0%). These regimens included conventional antibodies, checkpoint blockade, bispecific T-cell engagement, and cellular therapy-related strategies. The data describe trial design and do not compare the clinical value of individual combinations.

### 3.7 Safety reporting and trial-level toxicity patterns

Trial results were available for 253 of the 410 trials. Of these, 177 reported at least one usable toxicity outcome and were included in the safety analysis. A trial was considered safety-evaluable when the reported results identified an observed adverse event by event type, grade, frequency, or clinical consequence. Clinical consequences included toxicity-related treatment discontinuation, dose modification, and treatment-related death.

The other 76 trials reported efficacy only, used general safety statements without event-specific information, or described planned safety assessments without presenting observed events. Adverse-event definitions, follow-up periods, regimen composition, and reporting formats varied across studies. We therefore counted the number of trials reporting each toxicity category and did not pool patient-level incidence.

At least one Grade ≥3 adverse event was reported in 109 of 177 safety-evaluable trials (61.6%). Neutropenia was reported in 98 trials (55.4%). Infections were reported in 79 (44.6%), peripheral neuropathy in 69 (39.0%), anemia in 54 (30.5%), and thrombocytopenia in 52 (29.4%). Hepatotoxicity was reported in 33 trials (18.6%). Ocular toxicity was identified in seven trials, and pneumonitis or interstitial lung disease in five. Toxicity-related treatment discontinuation was reported in 22 trials (12.4%), dose modification in 10 (5.6%), and treatment-related death in 7 (4.0%).

Payload classes showed different reporting profiles. Among 128 safety-evaluable MMAE-containing trials, neutropenia was reported in 76 (59.4%), peripheral neuropathy in 65 (50.8%), and infection in 61 (47.7%). The calicheamicin group included 18 safety-evaluable trials. Hepatotoxicity was reported in 13 (72.2%), neutropenia in 10 (55.6%), and thrombocytopenia in 9 (50.0%).

Among 13 PBD-related trials, neutropenia and infection were each reported in 8 (61.5%). Anemia appeared in 6 (46.2%), and thrombocytopenia in 5 (38.5%). Ten maytansinoid-containing trials were safety-evaluable. Four reported thrombocytopenia, while three reported peripheral neuropathy and three reported ocular toxicity. Ocular toxicity was also reported in two of the three MMAF trials, although this estimate was based on a very small group.

Sixty-five safety-evaluable trials involved heavily pretreated patients or patients who had received at least two prior treatment lines. Thirty-nine of these trials (60.0%) reported at least one Grade ≥3 adverse event. Toxicity-related treatment discontinuation was identified in 11 (16.9%).

Cleavable linkers were used in 167 of 177 safety-evaluable trials (94.4%). The strong imbalance between linker groups, together with the close relationship between linker type, payload, and ADC program, prevented a reliable comparison of linker-specific off-target toxicity. Only three trials directly reported cumulative-dose or retreatment-related toxicity, which also limited the assessment of cumulative toxicity.

These trial-level reporting patterns do not represent direct payload-specific incidence or causality. Regimen composition, lymphoma subtype, prior treatment exposure, sample size, and adverse-event reporting differed across trials. In addition, 118 of the 177 safety-evaluable trials used combination regimens, making it difficult to attribute individual toxicities to the ADC or its payload alone.

## 4 Discussion

This systematic landscape analysis of 410 interventional trials shows that lymphoma ADC development is expanding but remains structurally concentrated and unevenly mature. Nearly half of the trials began between 2020 and 2025, yet 70.0% of the 50 unique ADC programs had not progressed beyond Phase I or Phase I/II. Late-phase activity was concentrated in DLBCL and cHL, while most other lymphoma subtypes remained dominated by exploratory studies. The field also relied heavily on a small group of targets, MMAE-based tubulin inhibition, and cleavable linkers. Accordingly, the central finding is not simply that trial activity has increased, but that expansion in trial numbers has outpaced diversification of ADC architecture and the generation of confirmatory evidence. This distinction is important because recent reviews describe a broad and increasingly sophisticated ADC pipeline, whereas the present trial-level analysis identifies where that apparent growth has—and has not—translated into mature lymphoma development (10, 21).

Clinical maturity was clustered around a small number of validated platforms. Brentuximab vedotin established CD30-directed ADC therapy in cHL and CD30-positive peripheral T-cell lymphomas, with randomized evidence supporting its use in frontline settings (11, 22). Polatuzumab vedotin subsequently moved CD79b-directed therapy into previously untreated DLBCL, and the five-year POLARIX analysis confirmed that the progression-free survival advantage was sustained, although the overall survival difference remained uncertain (13, 14). Loncastuximab tesirine demonstrated durable activity in heavily pretreated DLBCL, while ECHELON-3 extended brentuximab vedotin into a combination regimen for relapsed or refractory large B-cell lymphoma (15, 23). These milestones explain much of the observed concentration around CD30, CD79b, and CD19. They also show why success should be attributed to specific target–payload–linker–regimen combinations rather than generalized to ADCs as a uniform therapeutic class.

The design and endpoint findings further define the maturity gap. Single-arm studies accounted for 61.7% of the dataset, and only 18.5% of trials were randomized. Although randomization increased after 2010, its use remained close to one fifth of trials across the subsequent periods and did not show a sustained upward trend. Endpoint selection followed the expected phase structure: safety, dose-limiting toxicity, and maximum tolerated dose predominated in early development; ORR and CR/CMR dominated Phase II; and PFS was the leading Phase III endpoint, appearing in 21 of 34 Phase III trials. However, OS appeared in only six Phase III trials, and biomarker or correlative endpoints were exceptionally uncommon. This architecture can efficiently identify preliminary activity but is less suited to establishing comparative durability, optimal sequencing, or biologically defined benefit. Future late-phase trials should therefore combine clinically meaningful time-to-event endpoints with duration of response, patient-reported outcomes, treatment burden, and prospectively specified biomarkers. Randomized component-controlled designs will be particularly important for multi-agent regimens, in which the independent contribution of the ADC is otherwise difficult to determine.

Lymphoma subtype heterogeneity is central to interpreting these data. DLBCL and cHL together accounted for 41.6% of all trial–subtype pairs and contained most late-phase activity, whereas indolent B-cell lymphomas, mantle cell lymphoma, and several T-cell and rare lymphoma subtypes remained predominantly early phase. Target distributions followed known disease biology: CD30 was concentrated in cHL and mature T-cell lymphomas, CD79b in DLBCL, and CD19 and CD22 across a broader range of B-cell lymphomas. Thus, the numerical dominance of a target should not be interpreted independently of its disease context or eligible population. Among less established targets, ROR1 showed the clearest recent increase in trial activity, whereas CD70, CD37, and CD74 remained uncommon. These observations make ROR1 a rational emerging target for further evaluation, but they do not establish it as the most effective target. Moreover, a registry-based clinical landscape cannot rank preclinical candidates that have not yet entered registered trials. Prioritization should instead integrate subtype-specific surface density, internalization kinetics, antigen shedding, expression in essential normal tissues, and stability of expression after prior antigen-directed therapy (9, 21, 24).

The same caution applies to payload and linker development. Approximately four fifths of payload records involved tubulin inhibitors, and MMAE alone accounted for nearly three quarters; cleavable linkers predominated across every major subtype. This convergence reflects substantial clinical and manufacturing experience with auristatin-based, protease-cleavable platforms, but it also creates shared liabilities, including neuropathy, myelosuppression, off-target payload exposure, and cross-resistance. Topoisomerase I inhibitor payloads appeared in only six records, although their entry into late-phase development suggests a potentially important direction for diversification. Among the registered programs, SHR-A1912--a CD79b-directed ADC incorporating a cleavable linker and a topoisomerase I inhibitor payload—is a notable example of payload diversification because it has entered Phase III evaluation in relapsed or refractory DLBCL. However, no Phase III efficacy or safety results were available at the database cutoff, and its developmental stage should not be interpreted as evidence of clinical superiority. At present, this should be regarded as a development signal rather than evidence that topoisomerase I payloads are superior in lymphoma. More broadly, a cleavable linker is not intrinsically advantageous: its therapeutic value depends on plasma stability, intracellular release, payload membrane permeability, and the balance between bystander killing and off-target exposure. Site-specific conjugation, more homogeneous drug-to-antibody ratios, hydrophilic linkers, and payloads with mechanisms distinct from tubulin inhibition may widen the therapeutic window, but each innovation requires clinical rather than purely chemical validation (9, 24–27).

The inactive-trial analysis also argues against equating trial status with therapeutic success or failure. Among 92 terminated, closed, or temporarily closed trials, recruitment or feasibility problems were the most frequently reported reason, followed by the absence of a publicly specified reason and sponsor strategy or pipeline reprioritization. Insufficient efficacy and safety were each explicitly reported in only five records. These findings do not imply that biological failure was rare, because public explanations were incomplete and reasons were not independently adjudicated. Conversely, they show that operational feasibility, portfolio decisions, and reporting transparency materially shape the observed pipeline. The high numbers of inactive CD30-, CD79b-, and CD19-directed trials largely reflected the longer development history and greater trial exposure of these targets; without a program-at-risk denominator and time-to-event framework, these counts must not be interpreted as target-specific failure rates.

Safety remains a major determinant of whether early activity can become durable clinical benefit. Among 177 safety-evaluable trials, 109 reported at least one Grade 3 or higher adverse event, 98 reported neutropenia, 79 infection, and 69 peripheral neuropathy. Peripheral neuropathy was reported in 65 of 128 MMAE-containing trials, whereas hepatotoxicity was reported in 13 of 18 calicheamicin-containing trials. These are frequencies of trial-level reporting, not pooled patient-level incidences, and they cannot establish payload-specific causality. Regimens, populations, sample sizes, follow-up, and adverse-event definitions varied, and 118 safety-evaluable trials used combination therapy. Nevertheless, the patterns are biologically coherent and support platform-specific surveillance. They also underscore the need to report cumulative exposure, dose modification, discontinuation, and late toxicity more consistently, particularly in heavily pretreated patients and around transplantation. Only three trials directly reported cumulative-dose or retreatment-related toxicity, leaving an important evidence gap (25, 28, 29).

Resistance should likewise be interpreted as a multicomponent process. Potential mechanisms include loss or heterogeneous expression of the target antigen, impaired internalization, altered endosomal or lysosomal processing, drug-efflux transporters, reduced payload sensitivity, microenvironmental protection, and clonal selection after prior therapy (17, 18, 28, 29, 34, 35). The relative importance of these mechanisms is likely to differ between CD30-positive cHL, CD79b-positive DLBCL, and more heterogeneous CD19- or CD22-positive B-cell lymphomas. The present dataset did not directly measure resistance and therefore cannot assign mechanisms to individual inactive programs. Future trials should incorporate paired tissue or liquid biopsies, quantitative assessment of surface antigen and internalization, circulating tumor DNA, and assays of lysosomal function and payload sensitivity. Such studies would help distinguish resistance to the antibody target from resistance to the linker–payload platform and could guide target switching or payload switching at relapse.

The combination data provide a stronger immunologic context for lymphoma ADC development. At least one immune-based partner was used in 123 trials, including checkpoint inhibitors in 35 and bispecific antibodies or T-cell engagers in 31. ADCs may influence antitumor immunity through Fc-dependent effector functions, immunogenic cell death, release and presentation of tumor antigens, and payload-mediated bystander effects within the tumor microenvironment. These effects are not universal; they depend on antibody isotype, target biology, payload class, dose, and the immune composition of the disease. A payload that kills antigen-presenting or effector immune cells could counteract the intended synergy. The near absence of biomarker and correlative primary endpoints in the present dataset is therefore a missed opportunity. Immunotherapy-combination trials should prospectively evaluate immune-cell composition, antigen presentation, Fc function, cytokine changes, and clonal dynamics, while randomized designs should determine whether biological synergy translates into added clinical benefit rather than only added toxicity (30–32).

ADCs must also be positioned within a rapidly changing lymphoma treatment landscape. In advanced cHL, the S1826 trial showed superior progression-free survival with nivolumab plus AVD compared with brentuximab vedotin plus AVD, demonstrating that a validated ADC regimen may be displaced or repositioned when a more effective immune strategy emerges (33). In relapsed or refractory DLBCL, CAR-T-cell therapy, CD20×CD3 bispecific antibodies, transplantation, and off-the-shelf targeted combinations now compete across overlapping treatment lines; the Phase III STARGLO trial further established an overall survival benefit for glofitamab plus GemOx in transplant-ineligible disease (34). ADCs retain practical advantages because they are immediately available, familiar to administer, and can provide disease control without cell manufacturing. Their optimal role may therefore include frontline incorporation in biologically appropriate disease, an off-the-shelf option for patients ineligible for cellular therapy, or a bridge or salvage strategy when rapid treatment is needed. Sequencing should be individualized according to disease tempo, target preservation, prior toxicities, organ function, access, and the intended next therapy. There is not yet sufficient evidence for a universal sequence among ADCs, CAR-T cells, T-cell engagers, checkpoint inhibitors, small-molecule targeted agents, and transplantation (23, 33–35).Compared with TKIs, ADCs replace pathway inhibition with antigen-directed payload delivery; compared with TCEs, they do not depend primarily on T-cell redirection; and compared with CAR-T cells, they are immediately available and do not require individualized cell manufacturing. These distinctions support a complementary, subtype- and sequence-dependent role for ADCs rather than a universal hierarchy among these therapeutic platforms.

Experience with ADCs outside lymphoma offers two particularly relevant lessons. Gemtuzumab ozogamicin in acute myeloid leukemia showed that the therapeutic index of an ADC can change substantially with dose fractionation, patient selection, and combination context; it also established the importance of hepatotoxicity and transplant-related risk when using a calicheamicin platform (36). Trastuzumab deruxtecan showed how a cleavable linker, high drug-to-antibody ratio, topoisomerase I payload, and bystander activity can extend efficacy across heterogeneous antigen expression, but it also made interstitial lung disease a defining platform risk requiring proactive monitoring (9, 37). For lymphoma, these experiences argue that target selection alone is insufficient. Dose and schedule, drug-to-antibody ratio, linker stability, bystander activity, organ-specific toxicity, and compatibility with transplantation or immune therapy should be developed as an integrated package. Emerging topoisomerase I–based lymphoma ADCs should therefore include prospective pulmonary as well as hematologic safety assessment rather than assuming that a new payload will simply avoid MMAE-associated toxicity.

Several priorities follow from these findings. First, development should move from broad histology eligibility toward subtype- and biomarker-defined target selection, with repeat assessment of antigen expression after prior targeted therapy. Second, payload diversification should be pursued alongside pharmacologic evidence that the new mechanism can overcome established resistance without replacing neuropathy with an equally limiting toxicity. Third, linker and conjugation innovation should be evaluated using clinically interpretable pharmacokinetic, pharmacodynamic, and tissue-delivery measures. Fourth, early signals should transition more efficiently into randomized, comparator-based studies with PFS, OS, duration of response, patient-reported outcomes, and translational endpoints. Fifth, stronger academic–industry and cooperative-group partnerships may help move biologically novel programs beyond Phase II, while broader participation from underrepresented regions is needed to improve generalizability and access. The most defensible future direction is therefore not a single “best” receptor or payload, but a better matched target–payload–linker combination for a defined lymphoma subtype and treatment sequence (9, 24–27).

This study has several limitations. Trialtrove is a proprietary database, and although individual records were externally verified where possible, the original retrieval cannot be independently rerun without access to the platform. To improve analytical reproducibility, the complete trial-level dataset is provided in **Supplementary Table 1**, while the classification and coding rules are specified in the Methods and corresponding figure legends. Registry records may be incomplete, outdated, or inconsistent, particularly for proprietary payloads and linkers, treatment line, reasons for inactivity, and result reporting. Counts of trials, trial–subtype pairs, target–trial records, and sponsor–trial records represent different analytical units and should not be interpreted as patient exposure or development probabilities. Treatment setting could not be analyzed systematically because definitions were incomplete. The selected landmark studies provide clinical context but do not constitute a pooled efficacy analysis, and formal risk-of-bias or certainty-of-evidence assessments were not applicable to the landscape component. Safety analyses were limited to trial-level reporting, were vulnerable to selective reporting and combination-regimen confounding, and cannot estimate comparative adverse-event incidence. The protocol was not prospectively registered, and no formal inter-reviewer agreement statistic was calculated. Finally, the database cutoff means that subsequent trial updates, discontinuations, publications, and regulatory actions were not captured.

In conclusion, lymphoma ADC development has entered a phase of substantial clinical activity, but its maturity is concentrated in a small number of disease settings and platform designs. The strongest evidence supports specific regimens built around CD30, CD79b, and CD19 rather than the ADC class as a whole. Progress will depend on converting early-phase activity into randomized and biologically informed evidence, diversifying payloads and linkers without losing therapeutic index, and defining rational sequences with checkpoint blockade, bispecific antibodies, CAR-T-cell therapy, small-molecule targeted agents, and transplantation. A subtype-specific, biomarker-rich, and safety-conscious development strategy is more likely to produce durable clinical value than further expansion in trial numbers alone.

## Data Availability

The original contributions presented in the study are included in the article/**Supplementary Material**. Further inquiries can be directed to the corresponding authors.

## Glossary

ADC: Antibody–Drug Conjugate
ALCL: Anaplastic Large Cell Lymphoma
BCMA: B-Cell Maturation Antigen
CAR-T: Chimeric Antigen Receptor T-Cell Therapy
CD3: Cluster of Differentiation 3
CD19: Cluster of Differentiation 19
CD20: Cluster of Differentiation 20
CD22: Cluster of Differentiation 22
CD25: Cluster of Differentiation 25
CD30: Cluster of Differentiation 30
CD37: Cluster of Differentiation 37
CD70: Cluster of Differentiation 70
CD74: Cluster of Differentiation 74
CD79b: Cluster of Differentiation 79b
cHL: Classical Hodgkin Lymphoma
CI: Confidence Interval
CLL/SLL: Chronic Lymphocytic Leukemia/Small Lymphocytic Lymphoma
CMR: Complete Metabolic Response
CR: Complete Response
CTCL: Cutaneous T-Cell Lymphoma
DLBCL: Diffuse Large B-Cell Lymphoma
DLT: Dose-Limiting Toxicity
DM1: Mertansine
DM4: Ravtansine
DNA: Deoxyribonucleic Acid
DOR: Duration of Response
Fc: Fragment Crystallizable Region
FL: Follicular Lymphoma
G-CSF: Granulocyte Colony-Stimulating Factor
GGT: Gamma-Glutamyl Transferase
HGBL: High-Grade B-Cell Lymphoma
HER2: Human Epidermal Growth Factor Receptor 2
HR: Hazard Ratio
HSCT: Hematopoietic Stem Cell Transplantation
IPI: International Prognostic Index
LBCL: Large B-Cell Lymphoma
LBL: Lymphoblastic Lymphoma
LPL: Lymphoplasmacytic Lymphoma
MALT: Mucosa-Associated Lymphoid Tissue
MCL: Mantle Cell Lymphoma
MMAE: Monomethyl Auristatin E
MMAF: Monomethyl Auristatin F
MRD: Minimal Residual Disease
MTD: Maximum Tolerated Dose
MZL: Marginal Zone Lymphoma
NLPHL: Nodular Lymphocyte-Predominant Hodgkin Lymphoma
N-Ac-γ-Cal: N-Acetyl-γ-Calicheamicin
ORR: Objective Response Rate
OS: Overall Survival
PBD: Pyrrolobenzodiazepine
PFS: Progression-Free Survival
PK: Pharmacokinetics
PMBCL: Primary Mediastinal B-Cell Lymphoma
PRISMA: Preferred Reporting Items for Systematic Reviews and Meta-Analyses
PTCL: Peripheral T-Cell Lymphoma
RDE: Recommended Dose for Expansion
ROR1: Receptor Tyrosine Kinase-Like Orphan Receptor 1
RP2D: Recommended Phase II Dose
R/R: Relapsed or Refractory
RNA: Ribonucleic Acid
sALCL: Systemic Anaplastic Large Cell Lymphoma
TOP1i: Topoisomerase I Inhibitor
WM: Waldenström Macroglobulinemia
WM/LPL: Waldenström Macroglobulinemia/Lymphoplasmacytic Lymphoma
