# Supplementary material for: Antibody–drug conjugate development in lymphoma: a systematic clinical trial landscape analysis
Source: Front Immunol. 2026 Aug 19;17:1855476. doi: 10.3389/fimmu.2026.1855476 (PMC13487373; doi:10.3389/fimmu.2026.1855476)
Supplement: Supplementary file 1 [file Table3.docx]

**Completed for: Antibody–Drug Conjugate Development in Lymphoma: A Systematic Clinical Trial Landscape Analysis**

*Reporting context: PRISMA 2020–guided systematic clinical-trial landscape analysis; no meta-analysis was performed. This checklist maps the current report and does not imply that non-applicable or unperformed items were completed.*

Location note: line references correspond to the final line-numbered revised manuscript and should be updated only if pagination changes.

| **Section and Topic** | **Item #** | **Checklist item** | **Location where item is reported** |
| --- | --- | --- | --- |
| **TITLE** | | |  |
| **Title** | **1** | Identify the report as a systematic review. | **Reported —** Title, Lines 1–2 (identified as a ‘Systematic Clinical Trial Landscape Analysis’). |
| **ABSTRACT** | | |  |
| **Abstract** | **2** | See the PRISMA 2020 for Abstracts checklist. | **Reported —** Structured Abstract, Lines 17–43. |
| **INTRODUCTION** | | |  |
| **Rationale** | **3** | Describe the rationale for the review in the context of existing knowledge. | **Reported —** Introduction, Lines 50–88. |
| **Objectives** | **4** | Provide an explicit statement of the objective(s) or question(s) the review addresses. | **Reported —** Introduction, Lines 89–96. |
| **METHODS** | | |  |
| **Eligibility criteria** | **5** | Specify the inclusion and exclusion criteria for the review and how studies were grouped for the syntheses. | **Reported —** Methods Section 2.2, Lines 123–140; disease-subtype grouping is defined in Methods Section 2.3, Lines 153–160. |
| **Information sources** | **6** | Specify all databases, registers, websites, organisations, reference lists and other sources searched or consulted to identify studies. Specify the date when each source was last searched or consulted. | **Reported —** Trialtrove/Citeline source, search date, and cutoff are described in Methods Section 2.1, Lines 104–122; external verification sources are described at Lines 146–149. Trialtrove was the sole identification source; external sources were used for verification. |
| **Search strategy** | **7** | Present the full search strategies for all databases, registers and websites, including any filters and limits used. | **Reported —** The Trialtrove query, filters, phase limits, and date limits are presented in Methods Section 2.1, Lines 104–115. |
| **Selection process** | **8** | Specify the methods used to decide whether a study met the inclusion criteria of the review, including how many reviewers screened each record and each report retrieved, whether they worked independently, and if applicable, details of automation tools used in the process. | **Reported —** Methods Section 2.2, Lines 123–140. Two reviewers independently screened records; disagreements were resolved by consensus. No automation tool was used. |
| **Data collection process** | **9** | Specify the methods used to collect data from reports, including how many reviewers collected data from each report, whether they worked independently, any processes for obtaining or confirming data from study investigators, and if applicable, details of automation tools used in the process. | **Reported —** Methods Section 2.3, Lines 142–149. Two reviewers extracted the prespecified fields, and information was externally verified when available. No automation tool was used. |
| **Data items** | **10a** | List and define all outcomes for which data were sought. Specify whether all results that were compatible with each outcome domain in each study were sought (e.g. for all measures, time points, analyses), and if not, the methods used to decide which results to collect. | **Reported —** Primary-endpoint categories are defined in Methods Section 2.3, Lines 174–179; safety outcomes and eligibility for the safety analysis are defined in Methods Section 2.5, Lines 236–243. |
|  | **10b** | List and define all other variables for which data were sought (e.g. participant and intervention characteristics, funding sources). Describe any assumptions made about any missing or unclear information. | **Reported —** Trial, sponsor, subtype, ADC, target, payload, linker, regimen, and design variables are specified in Methods Section 2.3, Lines 142–199. Unconfirmed information was retained as ‘undisclosed,’ ‘not reported,’ or ‘unclear’ (Lines 136–149). |
| **Study risk of bias assessment** | **11** | Specify the methods used to assess risk of bias in the included studies, including details of the tool(s) used, how many reviewers assessed each study and whether they worked independently, and if applicable, details of automation tools used in the process. | **Not undertaken —** A formal study-level risk-of-bias assessment was not applied because this was a registered-trial landscape analysis and treatment effects were not synthesized; the rationale is given in Methods Section 2.6, Lines 248–252. |
| **Effect measures** | **12** | Specify for each outcome the effect measure(s) (e.g. risk ratio, mean difference) used in the synthesis or presentation of results. | **Not applicable —** No pooled treatment-effect measure was estimated. Analyses used counts and percentages, as stated in Methods Section 2.6, Lines 245–254; selected landmark outcomes are presented only for clinical context in Table 1 and Supplementary Table S2. |
| **Synthesis methods** | **13a** | Describe the processes used to decide which studies were eligible for each synthesis (e.g. tabulating the study intervention characteristics and comparing against the planned groups for each synthesis (item #5)). | **Reported —** Eligibility and grouping are described in Methods Section 2.2 (Lines 123–140); analytical units and denominators are defined in Methods Section 2.4 (Lines 200–221). |
|  | **13b** | Describe any methods required to prepare the data for presentation or synthesis, such as handling of missing summary statistics, or data conversions. | **Reported —** Standardization, consolidation, and classification procedures are described in Methods Section 2.3, Lines 141–199. Unresolved variables were not inferred; no outcome-statistic imputation or conversion was performed. |
|  | **13c** | Describe any methods used to tabulate or visually display results of individual studies and syntheses. | **Reported —** Tabulation and visualization methods are described in Methods Sections 2.4–2.6, Lines 200–254. Results are displayed in Figures 1–10, Table 1, and Supplementary Tables S1–S2. |
|  | **13d** | Describe any methods used to synthesize results and provide a rationale for the choice(s). If meta-analysis was performed, describe the model(s), method(s) to identify the presence and extent of statistical heterogeneity, and software package(s) used. | **Reported —** Descriptive synthesis used counts and percentages; no meta-analysis or pooled model was performed (Methods Section 2.6, Lines 245–254). |
|  | **13e** | Describe any methods used to explore possible causes of heterogeneity among study results (e.g. subgroup analysis, meta-regression). | **Not applicable —** No statistical synthesis or heterogeneity model was performed. Prespecified descriptive stratification by year, phase, subtype, target, payload, and linker is described in Methods Sections 2.4–2.6, Lines 200–254. |
|  | **13f** | Describe any sensitivity analyses conducted to assess robustness of the synthesized results. | **Not undertaken —** No sensitivity analysis was prespecified because no pooled or model-based estimate was generated (Methods Section 2.6, Lines 248–252). |
| **Reporting bias assessment** | **14** | Describe any methods used to assess risk of bias due to missing results in a synthesis (arising from reporting biases). | **Not undertaken —** Formal reporting-bias assessment was not performed. Registry records were included irrespective of result availability (Methods Section 2.6, Lines 248–251), and selective or incomplete reporting is acknowledged in the limitations (Lines 630–646). |
| **Certainty assessment** | **15** | Describe any methods used to assess certainty (or confidence) in the body of evidence for an outcome. | **Not undertaken —** No certainty-of-evidence grading was performed because comparative treatment effects were not synthesized (Methods Section 2.6, Lines 248–252). |
| **RESULTS** | | |  |
| **Study selection** | **16a** | Describe the results of the search and selection process, from the number of records identified in the search to the number of studies included in the review, ideally using a flow diagram. | **Reported —** Results Section 3.1, Lines 256–260, and the PRISMA-based flow diagram and legend, Figure 1, Lines 799–812. |
|  | **16b** | Cite studies that might appear to meet the inclusion criteria, but which were excluded, and explain why they were excluded. | **Partly reported —** Aggregate exclusion reasons and counts are reported in Results Section 3.1 (Lines 256–260) and Figure 1 (Lines 799–812). A record-level list of apparently eligible excluded studies is not provided. |
| **Study characteristics** | **17** | Cite each included study and present its characteristics. | **Reported —** Principal characteristics of all included trials are provided in Supplementary Table S1; selected landmark and contemporary trials are detailed in Table 1 (Lines 907–916) and Supplementary Table S2. Aggregate characteristics are reported in Results, Lines 256–471. |
| **Risk of bias in studies** | **18** | Present assessments of risk of bias for each included study. | **Not undertaken —** No study-level risk-of-bias assessment was performed; see Item 11 and Methods Section 2.6, Lines 248–252. |
| **Results of individual studies** | **19** | For all outcomes, present, for each study: (a) summary statistics for each group (where appropriate) and (b) an effect estimate and its precision (e.g. confidence/credible interval), ideally using structured tables or plots. | **Partly applicable —** Trial-level efficacy and safety outcomes are reported for selected landmark or advanced trials in Table 1 and Supplementary Table S2. For the full landscape, outcomes were summarized as trial-level reporting frequencies and were not pooled. |
| **Results of syntheses** | **20a** | For each synthesis, briefly summarise the characteristics and risk of bias among contributing studies. | **Reported —** The contributing trials are summarized by phase, design, subtype, endpoint, target, platform, status, and safety-reporting availability in Results, Lines 256–471. Formal risk-of-bias assessments were not performed. |
|  | **20b** | Present results of all statistical syntheses conducted. If meta-analysis was done, present for each the summary estimate and its precision (e.g. confidence/credible interval) and measures of statistical heterogeneity. If comparing groups, describe the direction of the effect. | **Not applicable —** No statistical synthesis or meta-analysis was conducted (Methods Section 2.6, Lines 245–254). |
|  | **20c** | Present results of all investigations of possible causes of heterogeneity among study results. | **Not applicable —** No statistical investigation of heterogeneity was conducted; descriptive phase- and subtype-stratified results are presented in Results Sections 3.2–3.5, Lines 284–419. |
|  | **20d** | Present results of all sensitivity analyses conducted to assess the robustness of the synthesized results. | **Not applicable —** No sensitivity analyses were conducted because no pooled estimate was generated (Methods Section 2.6, Lines 248–252). |
| **Reporting biases** | **21** | Present assessments of risk of bias due to missing results (arising from reporting biases) for each synthesis assessed. | **Not undertaken —** No formal reporting-bias assessment was performed. Limitations arising from incomplete and selective registry or result reporting are discussed at Lines 630–646. |
| **Certainty of evidence** | **22** | Present assessments of certainty (or confidence) in the body of evidence for each outcome assessed. | **Not undertaken —** No certainty-of-evidence assessment was performed; see Methods Section 2.6, Lines 248–252. |
| **DISCUSSION** | | |  |
| **Discussion** | **23a** | Provide a general interpretation of the results in the context of other evidence. | **Reported —** Discussion, Lines 472–629, interprets trial maturity, subtype heterogeneity, platform concentration, inactive development, safety, resistance, immune combinations, and treatment sequencing in the context of other evidence. |
|  | **23b** | Discuss any limitations of the evidence included in the review. | **Reported —** Limitations of the available evidence, including heterogeneous safety reporting and absence of patient-level denominators, are discussed at Lines 550–561 and 630–646. |
|  | **23c** | Discuss any limitations of the review processes used. | **Reported —** Limitations of the review process, including reliance on a proprietary single database, external-verification constraints, cutoff effects, and lack of pooled, risk-of-bias, and certainty analyses, are discussed at Lines 630–646. |
|  | **23d** | Discuss implications of the results for practice, policy, and future research. | **Reported —** Implications for trial design, biomarker selection, safety reporting, immune or cellular-therapy sequencing, and future research are discussed at Lines 495–507 and 573–629. |
| **OTHER INFORMATION** | | |  |
| **Registration and protocol** | **24a** | Provide registration information for the review, including register name and registration number, or state that the review was not registered. | **Reported —** The review was not prospectively registered (Methods Section 2.1, Lines 116–122). |
|  | **24b** | Indicate where the review protocol can be accessed, or state that a protocol was not prepared. | **Not publicly available —** No registered or publicly accessible protocol is available; the manuscript states that the protocol was not prospectively registered (Lines 116–122). |
|  | **24c** | Describe and explain any amendments to information provided at registration or in the protocol. | **Not applicable —** There was no registered or publicly accessible protocol against which amendments could be reported. |
| **Support** | **25** | Describe sources of financial or non-financial support for the review, and the role of the funders or sponsors in the review. | **Reported —** Funding: None (Line 952); therefore, no funder or sponsor role applied. |
| **Competing interests** | **26** | Declare any competing interests of review authors. | **Reported —** Conflict of Interest statement, Lines 939–941. |
| **Availability of data, code and other materials** | **27** | Report which of the following are publicly available and where they can be found: template data collection forms; data extracted from included studies; data used for all analyses; analytic code; any other materials used in the review. | **Partly reported —** Data Availability Statement, Lines 953–959. Supplementary Table S1 provides trial identifiers and principal extracted variables for the 410 included trials, and Supplementary Table S2 provides details on selected landmark trials. The supplementary files do not reproduce the proprietary Trialtrove records or database interface; a data-collection form and analytic code are not listed as publicly available. |

*From:*  Page MJ, McKenzie JE, Bossuyt PM, Boutron I, Hoffmann TC, Mulrow CD, et al. The PRISMA 2020 statement: an updated guideline for reporting systematic reviews. BMJ 2021;372:n71. doi: 10.1136/bmj.n71. This work is licensed under CC BY 4.0. To view a copy of this license, visit <https://creativecommons.org/licenses/by/4.0/>
